# Topology and pore-surface engineering of a Zr-MOF for ethylene purification and propylene recovery from MTO products

**DOI:** 10.1039/d6sc05534f

**Published:** 2026-10-01

**Authors:** Ran Zheng, Qiang Zhang, Lulu Wang, Mingwei Wan, Zhaoquan Yao, Tongliang Hu, Jian Xu, Xian-He Bu

**Affiliations:** a School of Materials Science and Engineering, Smart Sensing Interdisciplinary Science Center, TKL of Metal and Molecule-Based Material Chemistry, Academy for Advanced Interdisciplinary Studies, Nankai University Tianjin 300350 China jxu@nankai.edu.cn; b School of Chemistry and Chemical Engineering, Tianjin University of Technology Tianjin 300384 China; c State Key Laboratory of Elemento-Organic Chemistry, Frontiers Science Center for New Organic Matter, Collaborative Innovation Center of Chemical Science and Engineering (Tianjin), Nankai University Tianjin 300071 China

## Abstract

Efficient separation of light hydrocarbons from methanol-to-olefins (MTO) product streams, typically consisting of ethylene (C_2_H_4_), propylene (C_3_H_6_), and ethane (C_2_H_6_), is pivotal for sustainable olefin production but remains challenged by the trade-off between selectivity and capacity. Herein, we report a highly robust zirconium-based metal–organic framework, NKU-400, constructed from 12-connected Zr_6_ clusters and tetratopic carboxylate ligands, featuring a unique (4,4,12)-connected topology with aromatic-rich and N/O-functionalized pore surfaces. The tailored pore environment affords a pronounced thermodynamic preference for C_3_H_6_ over C_2_H_4_ through synergistic pore confinement and multiple C–H⋯π/O/N interactions, while a more modest discrimination between C_2_H_6_ and C_2_H_4_ develops at finite loading. Consequently, NKU-400 exhibits high adsorption capacities for C_3_H_6_ (6.01 mmol g^−1^) and C_2_H_6_ (4.29 mmol g^−1^), together with high C_3_H_6_/C_2_H_4_ IAST selectivities of 9.83–10.67 at 298 K. Dynamic breakthrough experiments further demonstrate highly effective binary C_3_H_6_/C_2_H_4_ separation, delivering polymer-grade C_2_H_4_ (≥99.95%) with a productivity of 94.45 L(STP) kg^−1^ and enabling the subsequent recovery of high-purity C_3_H_6_ (≥99.5%) with a productivity of 77.62 L(STP) kg^−1^ from an equimolar mixture. Furthermore, NKU-400 enables the one-step purification of polymer-grade C_2_H_4_ from ternary C_3_H_6_/C_2_H_6_/C_2_H_4_ mixtures, followed by the recovery of high-purity C_3_H_6_ with a productivity of 59.72 L(STP) kg^−1^ within a single adsorption–desorption cycle. Combined with its excellent chemical robustness, these results establish NKU-400 as a promising adsorbent for the purification of MTO-derived light hydrocarbons and highlight the effectiveness of integrating topology design with pore-surface engineering for challenging hydrocarbon separations.

## Abstract 2

Robust NKU-400 features aromatic-rich, N/O-functionalized channels that enable one-step purification of C_2_H_4_ to polymer-grade purity and subsequent recovery of high-purity C_3_H_6_ from multicomponent MTO streams.

## Graphical abstract

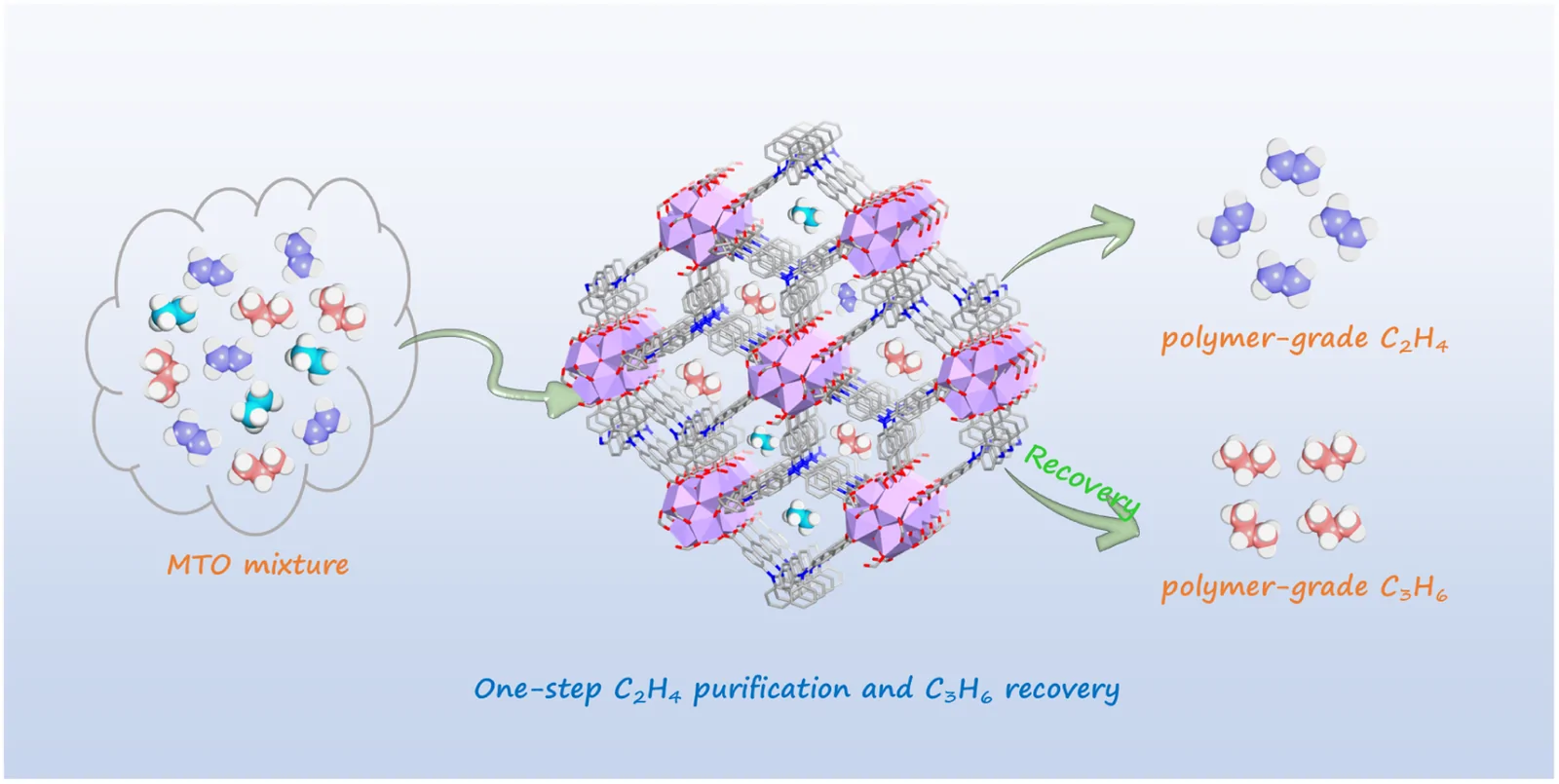

## 1 Introduction

Ethylene (C_2_H_4_) and propylene (C_3_H_6_) are fundamental feedstocks in the modern petrochemical industry and are extensively employed in the production of bulk chemicals, polymers, and functional materials.^1–4^ While naphtha steam cracking remains the dominant technology for olefin production, the methanol-to-olefins (MTO) process has emerged as a more sustainable alternative that can alleviate dependence on petroleum resources.^5–7^ However, the MTO process typically produces a mixed product stream containing C_2_H_4_ (51.1%) and C_3_H_6_ (20.9%), along with ethane (C_2_H_6_) impurities (6–10%), which requires efficient separation to obtain polymer-grade purity.^8–10^ Conventional cryogenic distillation for obtaining polymer-grade olefins is notoriously energy-intensive and economically demanding, driving the search for alternative separation technologies.^11,12^

Adsorptive separation using porous solids, particularly metal–organic frameworks (MOFs), has attracted extensive attention as an energy-efficient strategy.^13–15^ MOFs are crystalline porous networks formed by self-assembly of metal ions or clusters with multitopic organic linkers, and they offer unparalleled structural and chemical tunability.^16–18^ This versatility allows precise control over pore geometry and surface chemistry, enabling molecular-level recognition in gas separation. To date, numerous MOFs have been developed for binary separations such as C_2_H_6_/C_2_H_4_ and C_3_H_6_/C_2_H_4_.^19–22^ In contrast, materials capable of directly and simultaneously recovering polymer-grade C_2_H_4_ and C_3_H_6_ from ternary C_3_H_6_/C_2_H_6_/C_2_H_4_ mixtures are highly desirable, as they would simplify process flowsheets and significantly reduce energy consumption. The molecular similarity of these hydrocarbons in size and physicochemical properties makes their discrimination intrinsically difficult.

Generally, MOFs bearing open metal sites (OMSs) can preferentially adsorb C_3_H_6_ over C_2_H_4_ by exploiting the higher gas-phase acidity of C_3_H_6_.^23–25^ However, OMSs also tend to interact more strongly with C_2_H_4_ than with C_2_H_6_, making the preferential adsorption of C_2_H_6_ over C_2_H_4_ difficult to achieve.^26–28^ To simultaneously favor the adsorption of C_3_H_6_ and C_2_H_6_ over C_2_H_4_, two complementary pore-engineering principles can be considered. The first exploits differences in molecular polarizability (C_3_H_6_: 62.6 × 10^−25^ cm^3^; C_2_H_6_: 44.7 × 10^−25^ cm^3^; C_2_H_4_: 42.52 × 10^−25^ cm^3^) by constructing relatively nonpolar, aromatic-rich pore environments. Such pore surfaces can enhance dispersion and C–H⋯π interactions with the larger and more polarizable C_3_H_6_ and C_2_H_6_ molecules.^29–31^ The second involves the introduction of appropriately positioned nonmetallic functional sites, such as N and O atoms, which can provide multiple C–H⋯N/O interactions and further strengthen host–guest recognition.^32–34^ Integrating these strategies with precise control over pore size and pore polarity offers a promising route to balancing adsorption capacity and selectivity for the one-step production of polymer-grade C_2_H_4_. Moreover, the preferentially adsorbed C_3_H_6_ can subsequently be recovered at high purity through desorption, further enhancing the overall utility of the separation process.

Guided by these considerations, we designed and synthesized a novel zirconium-based MOF, named NKU-400, featuring a unique (4,4,12)-connected topology and well-defined one-dimensional channels lined with π-rich aromatic moieties and well-positioned N/O sites. Such a tailored pore environment integrates pore confinement with multiple weak host–guest interactions, including C–H⋯π/O/N interactions, thereby providing pronounced discrimination of C_3_H_6_ over C_2_H_4_ while enabling more moderate differentiation between C_2_H_6_ and C_2_H_4_ at finite loading. Benefiting from the combination of high adsorption capacity and selective molecular recognition, NKU-400 exhibits highly effective binary C_3_H_6_/C_2_H_4_ separation and further enables the one-step purification of polymer-grade C_2_H_4_ from ternary C_3_H_6_/C_2_H_6_/C_2_H_4_ mixtures, followed by the recovery of high-purity C_3_H_6_ within a single adsorption–desorption cycle. These results highlight the effectiveness of integrating topology design with pore-surface engineering for the separation and recovery of valuable olefins from complex MTO product streams.

## 2 Results and discussion

### 2.1 Structural analysis

Guided by the aforementioned design principle, we successfully constructed a novel zirconium-based metal–organic framework, denoted as NKU-400, *via* a solvothermal reaction of ZrCl_4_ and *N*,*N*,*N*′,*N*′-tetrakis(4-carboxyphenyl)-1,4-phenylenediamine (H_4_TCPPDA) in *N*,*N*-dimethylformamide (DMF) with benzoic acid (BA) as the modulator. Light-yellow truncated octahedral crystals were obtained (Fig. S1), and their structure was determined by single-crystal X-ray diffraction (SCXRD). Structural analysis shows that NKU-400, with the formula Zr_6_(µ_3_-O)_4_(µ_3_-OH)_4_(TCPPDA)_3_(H_2_O)_2_, crystallizes in the monoclinic space group *C*2/*c* with unit-cell parameters *a* = 22.2447 Å, *b* = 27.3434 Å, *c* = 30.8176 Å and *β* = 100.980° (Table S1).

In particular, the framework is built from {Zr_6_(µ_3_-O)_4_(µ_3_-OH)_4_} clusters, each coordinated by twelve carboxylate groups from twelve TCPPDA^4−^ ligands and two terminal water molecules. Among these carboxylates, ten adopt a bridging bidentate mode across two Zr atoms, while the remaining two adopt a monodentate mode on a single Zr atom (Fig. 1a). The resulting coordination polyhedron of the Zr_6_ cluster can be described as an octadecahedron, which differs from the cuboctahedral geometry typical of **ftw** net or the icosahedral geometry found in the **ith** series^35–38^ (Fig. S2). Moreover, the TCPPDA^4−^ ligand exhibits two distinct geometric conformations (Type I and Type II) arising from rotational flexibility around the C–N bonds, leading to different dihedral angles between the central and peripheral phenyl rings (68.1°/66.4°/71.7°/60.7° for Type I; 66.7°/76.3°/67.6°/76.9° for Type II, Table S2). This conformational diversity results in an overall low *C*_1_ symmetry in a tetrahedral arrangement (Fig. 1b). Each Zr_6_ cluster connects to eight Type I and four Type II ligands, forming a 12-connected node (Fig. S3). Topologically, the unique coordination of the Zr_6_ clusters combined with the two ligand conformations gives rise to a previously unreported (4,4,12)-connected net with the point symbol of {4^26^·6^32^·8^8^}{4^6^}_3_ (Fig. 1c and d). The resulting three-dimensional framework features uniform diamond-shaped one-dimensional channels with a pore aperture of 10.0 Å along the *c* axis (Fig. 1e). The total guest-accessible volume calculated by PLATON accounts for 59.5% of the unit-cell volume, providing ample space for gas adsorption.

**Fig. 1:**
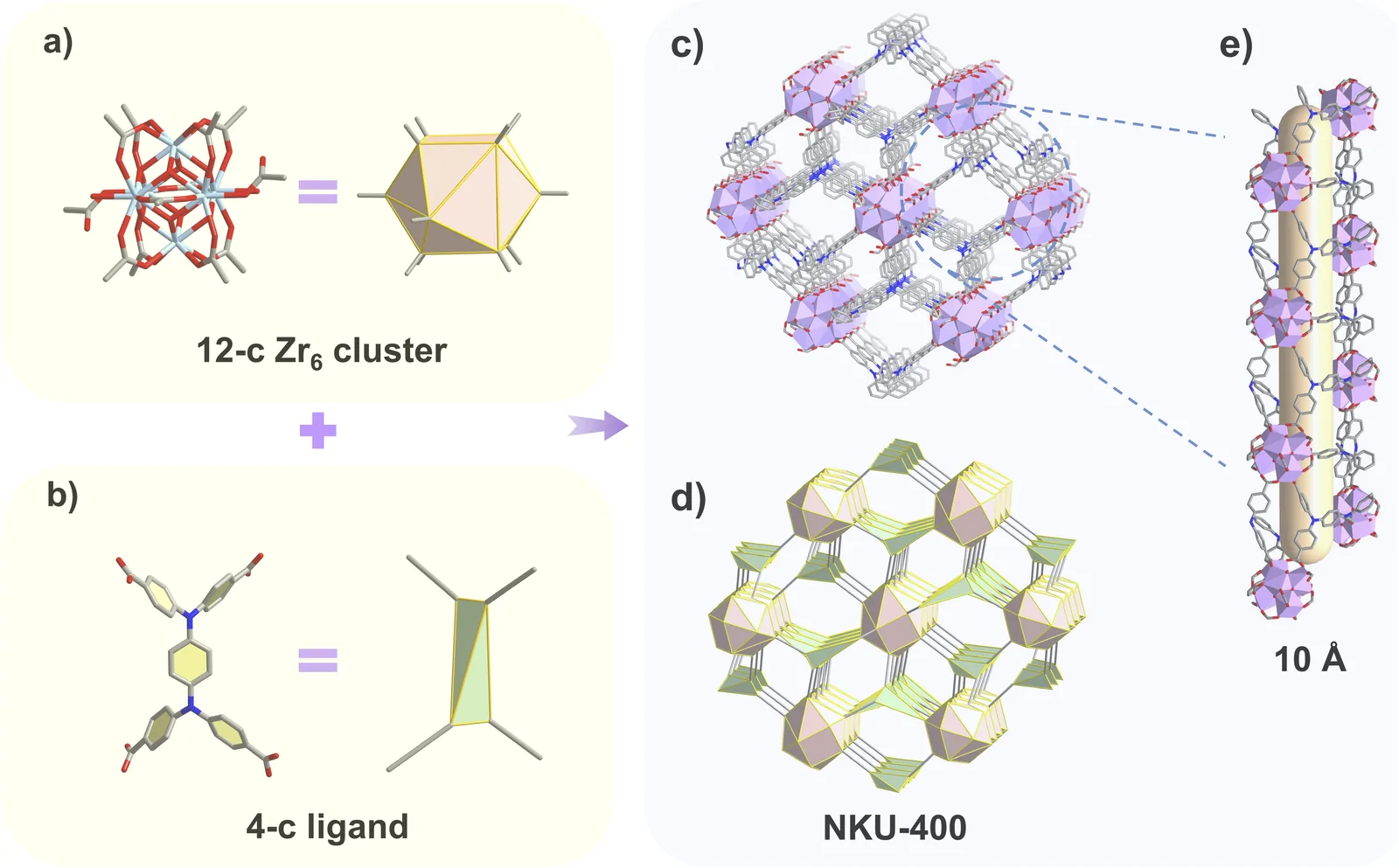
Schematic representation of the assembly of (a) the 12-connected Zr_6_ cluster and (b) the 4-connected ligand to generate (c) NKU-400; (d) topologically simplified net of NKU-400; (e) the view of the uniform 1D channel. Zr, C, N and O are represented by cyan, gray, blue and red, respectively. H atoms are omitted for clarity.

### 2.2 Structural characterization

The phase purity of as-synthesized NKU-400 was confirmed by the excellent agreement between the experimental and simulated powder X-ray diffraction (PXRD) patterns (Fig. S4). Attributed to the strong Zr–O bonds and high connectivity of the Zr_6_O_8_ clusters, the framework exhibits outstanding structural stability. Thermogravimetric analysis (TGA) reveals that the framework does not decompose until a temperature of 450 °C (Fig. S5). To evaluate its chemical stability, the pristine sample was immersed in various media, including organic solvents, water, concentrated HCl aqueous solution, and NaOH aqueous solution (pH = 11) for 48 h. The PXRD patterns collected after these treatments show no loss of crystallinity or structural degradation, confirming the remarkable robustness of NKU-400 under harsh chemical conditions (Fig. 2a, S6 and S7).

**Fig. 2:**
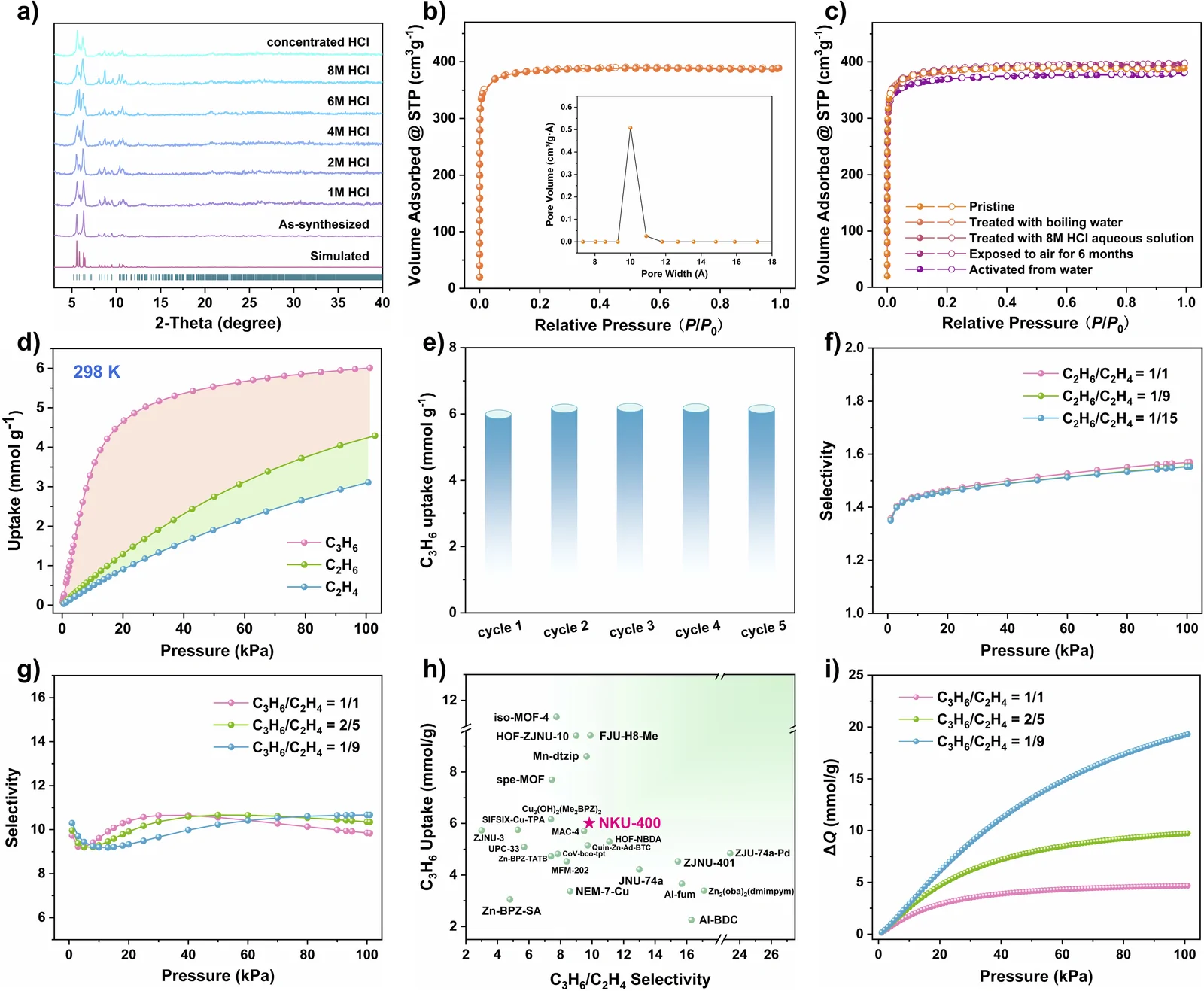
(a) PXRD patterns of NKU-400 after immersion in aqueous HCl solutions of different concentrations for 48 h. (b) N_2_ adsorption–desorption isotherm of NKU-400 at 77 K and the corresponding pore size distribution (inset). (c) N_2_ adsorption–desorption isotherms at 77 K of the pristine MOF and samples treated under different conditions. (d) Single-component adsorption isotherms of NKU-400 for C_3_H_6_, C_2_H_6_ and C_2_H_4_ at 298 K; (e) cycling performance of NKU-400 for C_3_H_6_ adsorption at 298 K over five consecutive adsorption–desorption cycles. (f and g) Predicted IAST selectivity for C_2_H_6_/C_2_H_4_ (1/1, 1/9 and 1/15, v/v) and C_3_H_6_/C_2_H_4_ (1/1, 2/5 and 1/9, v/v) mixtures at 298 K, respectively. (h) Comparison of C_3_H_6_/C_2_H_4_ selectivity (1/1, v/v) and C_3_H_6_ uptake capacity at 298 K and 1 bar for NKU-400 and benchmark adsorbents. (i) Separation potentials of NKU-400 for C_3_H_6_/C_2_H_4_ mixtures at 298 K.

In order to investigate the permanent porosity of NKU-400, the sample was solvent-exchanged with acetone and activated under dynamic vacuum at 150 °C for 12 h. The PXRD pattern of the activated sample remained consistent with that of the as-synthesized material, indicating preservation of the framework integrity upon desolvation (Fig. S4). N_2_ adsorption–desorption measurements at 77 K yielded a fully reversible Type-I isotherm, characteristic of microporous materials, with a sharp uptake at low relative pressure (*P*/*P*_0_ ≈ 0.01) (Fig. 2b). Particularly, the N_2_ uptake reached 388 cm^3^ g^−1^ at *P*/*P*_0_ = 0.90, close to the theoretical value of 430 cm^3^ g^−1^ estimated from the solvent-accessible volume (59.5% of the unit cell) calculated by PLATON and the crystallographic density (0.893 g cm^−3^, Table S1). The Brunauer–Emmett–Teller (BET) surface area and total pore volume were calculated to be 1174 m^2^ g^−1^ and 0.598 cm^3^ g^−1^, respectively. Nonlocal density functional theory (NLDFT) analysis of the adsorption branch indicated a narrow pore size distribution centered at 10 Å (Fig. 2b, inset), in good agreement with the pore dimensions derived from single-crystal structure after accounting for van der Waals radii.

To further assess stability under challenging environments, N_2_ sorption isotherms were measured for samples treated with boiling water, treated with 8 M HCl aqueous solution for 48 h, exposed to air for 6 months and activated directly from water. Remarkably, all treated samples exhibited adsorption capacities nearly identical to that of the pristine material (Fig. 2c), underscoring the ultrahigh stability of NKU-400. Combined with its well-defined microporous structure, these results highlight the strong potential of NKU-400 for applications in gas adsorption and separation.

### 2.3 Gas adsorption and separation properties

The combination of permanent porosity, optimal pore dimensions, and aromatic-rich pore surfaces in NKU-400 prompted a systematic investigation of its performance in separating MTO products. Single-component adsorption isotherms of C_3_H_6_, C_2_H_4_ and C_2_H_6_ were measured at 273, 298, and 308 K. As shown in Fig. 2d, S8 and S9, all isotherms exhibit Type-I behavior. The steep uptake of C_3_H_6_ in the low-pressure region indicates a stronger adsorption affinity compared to C_2_H_6_ and C_2_H_4_, following the trend C_3_H_6_ > C_2_H_6_ > C_2_H_4_. Specifically, NKU-400 exhibits high adsorption capacities of 6.61/6.01/5.60 mmol g^−1^ for C_3_H_6_ and 5.75/4.29/3.42 mmol g^−1^ for C_2_H_6_ at 273/298/308 K, which are significantly higher than those for C_2_H_4_ (4.86/3.11/2.59 mmol g^−1^). To complement the gravimetric adsorption capacity, the volumetric C_3_H_6_ uptake of NKU-400 was estimated using its crystallographic density (0.893 g cm^−3^). At 298 K and 1 bar, NKU-400 affords a volumetric C_3_H_6_ uptake of 120.15 cm^3^(STP) cm^−3^ (5.37 mmol cm^−3^), which is competitive with representative porous adsorbents under comparable conditions (Table S3). The higher uptakes of C_3_H_6_ and C_2_H_6_ relative to C_2_H_4_ can be associated with their larger molecular polarizabilities and more extensive contact with the aromatic/N/O-functionalized pore surface, although the relative contributions of these effects vary with adsorption loading.^39–41^

To gain deeper insight into the adsorption behavior, the isosteric heats of adsorption (*Q*_st_) were recalculated by simultaneously fitting the adsorption isotherms measured at 273, 298, and 308 K using the virial method (Fig. S10–S13 and Table S4). The resulting zero-coverage *Q*_st_ values are 29.65 kJ mol^−1^ for C_3_H_6_, 23.78 kJ mol^−1^ for C_2_H_6_, and 23.74 kJ mol^−1^ for C_2_H_4_. The substantially higher value for C_3_H_6_ indicates a pronounced thermodynamic preference for C_3_H_6_ at infinite dilution, whereas the nearly identical values for C_2_H_6_ and C_2_H_4_ indicate only limited initial enthalpic discrimination between these two adsorbates.

Interestingly, the *Q*_st_ profiles exhibit a nonmonotonic dependence on adsorption loading. At low coverage, adsorbate molecules preferentially occupy the energetically most favorable adsorption environments, where relatively strong host–guest interactions dominate. As these high-affinity environments become progressively occupied, subsequently adsorbed molecules populate less favorable regions of the pore, resulting in an initial decrease in *Q*_st_. At higher loadings, the intermolecular distances between adsorbed molecules decrease within the confined channels, and adsorbate–adsorbate interactions, particularly dispersion interactions, are expected to become increasingly important. The resulting additional stabilization can reasonably account for the subsequent increase in *Q*_st_. This high-loading upturn is most pronounced for C_3_H_6_, which possesses the highest molecular polarizability and also reaches the highest adsorption loading among the three hydrocarbons, favoring stronger cumulative intermolecular interactions upon dense pore filling. In comparison, the increase is less pronounced for C_2_H_6_ and weakest for C_2_H_4_. Notably, although C_2_H_6_ and C_2_H_4_ exhibit nearly identical *Q*_st_ values at zero coverage, their profiles progressively diverge with increasing loading, with C_2_H_6_ generally maintaining higher *Q*_st_ values in the finite-loading region. This behavior suggests that the modest C_2_H_6_/C_2_H_4_ discrimination develops progressively during pore filling rather than originating from a pronounced difference in their strongest adsorption sites at infinite dilution.

Among reported MOFs for C_2_H_4_ purification, the moderate *Q*_st_ values of C_2_H_6_ and C_3_H_6_ on NKU-400 suggest facile adsorbent regeneration with low energy input (Fig. S14 and S15). In addition, cyclic adsorption measurements for C_3_H_6_ at 298 K reveal nearly unchanged uptake capacities over five consecutive adsorption–desorption cycles (Fig. 2e), further confirming the excellent regenerability and adsorption stability of NKU-400. Furthermore, water vapor adsorption measurements confirm the hydrophobic nature of the pore surface. The isotherm displays a typical S-shaped profile with a hysteresis loop and an inflection point at relatively high relative humidity (Fig. S16), indicating weak water-binding affinity, which can be attributed to the abundance of aromatic rings in the framework.

To further evaluate the separation performance, ideal adsorbed solution theory (IAST)^42^ was employed to predict selectivity in binary mixtures (Fig. S17–S19 and Table S5). As illustrated in Fig. 2f and S20, NKU-400 exhibits modest yet stable C_2_H_6_/C_2_H_4_ selectivities of 1.55–1.64 over a range of temperatures and gas compositions (1/1, 1/9, and 1/15 v/v). Under equimolar conditions (1/1, v/v), although the C_2_H_6_/C_2_H_4_ selectivity of NKU-400 is lower than those of top-performing materials such as Fe_2_(O_2_)(dobdc) (4.4),^43^ Cu(Qc)_2_ (3.4),^44^ and NKMOF-8-Br (2.65),^45^ it remains superior or comparable to those of several C_2_H_6_-selective MOFs, including LIFM-62 (1.4),^46^ MIL-53-BPDC (1.5),^47^ and CPM-734c (1.44).^48^

More notably, NKU-400 achieves exceptional C_3_H_6_/C_2_H_4_ IAST selectivity of 9.83, 10.34, and 10.67 at 298 K for mixtures with compositions of 1/1, 2/5, and 1/9 (v/v), respectively (Fig. 2g and S21). Under equimolar conditions (1/1, v/v), the selectivity of NKU-400 (9.83) surpasses those of several benchmark materials, including MAC-4 (9.5),^49^ spe-MOF (7.45),^50^ NEM-7-Cu (8.6),^51^ and HOF-ZJNU-3 (3.0)^52^ (Fig. 2h and Table S6). To provide a more rigorous evaluation of the actual separation efficiency, the separation potential (Δ*Q*), a combined metric of selectivity and capacity proposed by Krishna, was calculated to estimate the maximum C_2_H_4_ productivity.^53,54^ The corresponding Δ*Q* values for the 1/1, 2/5, and 1/9 C_3_H_6_/C_2_H_4_ mixtures reached 4.66, 9.73, and 19.31 mmol g^−1^ at 298 K, respectively (Fig. 2i). Comparative analysis unequivocally demonstrates that NKU-400 outperforms most reported adsorbents in terms of overall separation potential (Fig. S22). This validates its superior separation capability, which is driven by an optimal balance between capacity and selectivity rather than maximizing one at the expense of the other. Consequently, NKU-400 successfully overcomes the conventional trade-off between uptake and selectivity. Together with its high adsorption capacities for C_3_H_6_ and C_2_H_6_, competitive selectivity, and moderate adsorption enthalpies, these findings highlight the immense potential of NKU-400 for the efficient one-step purification of C_2_H_4_ from ternary C_3_H_6_/C_2_H_4_/C_2_H_6_ mixtures in MTO process.

### 2.4 Breakthrough experiments

To probe mass-transfer behavior within the approximately 10 Å one-dimensional channels of NKU-400, apparent mass-transfer-zone (MTZ) lengths were derived from the single-component breakthrough curves (Fig. S23 and Table S7). The normalized MTZ lengths were 15.4%, 15.3%, and 29.5% for C_2_H_4_, C_2_H_6_, and C_3_H_6_, respectively, revealing comparable breakthrough-front broadening for the two C_2_ hydrocarbons but a broader front for C_3_H_6_. The latter is consistent with greater overall mass-transfer resistance for the larger C_3_H_6_ molecule, although the apparent MTZ parameter reflects combined equilibrium and transport effects rather than intrinsic diffusivity alone.

Binary breakthrough experiments were subsequently conducted with C_2_H_6_/C_2_H_4_ mixtures with varying compositions. As illustrated in Fig. 3a, NKU-400 achieved efficient separation in all cases, with C_2_H_4_ eluting first from the column to afford high-purity C_2_H_4_ (≥99.95%), while C_2_H_6_ was preferentially retained. Unless otherwise specified, all volumetric productivities derived from the breakthrough experiments are reported on an STP basis (273.15 K and 1 atm). The corresponding high-purity C_2_H_4_ productivities were estimated to be 0.94, 2.37, and 4.26 L(STP) kg^−1^ for the 1/1, 1/9 and 1/15 C_2_H_6_/C_2_H_4_ mixtures at 298 K, respectively. Moreover, NKU-400 maintained consistent separation performance over five consecutive breakthrough cycles and could be completely regenerated by Ar purging under ambient conditions (Fig. S24).

**Fig. 3:**
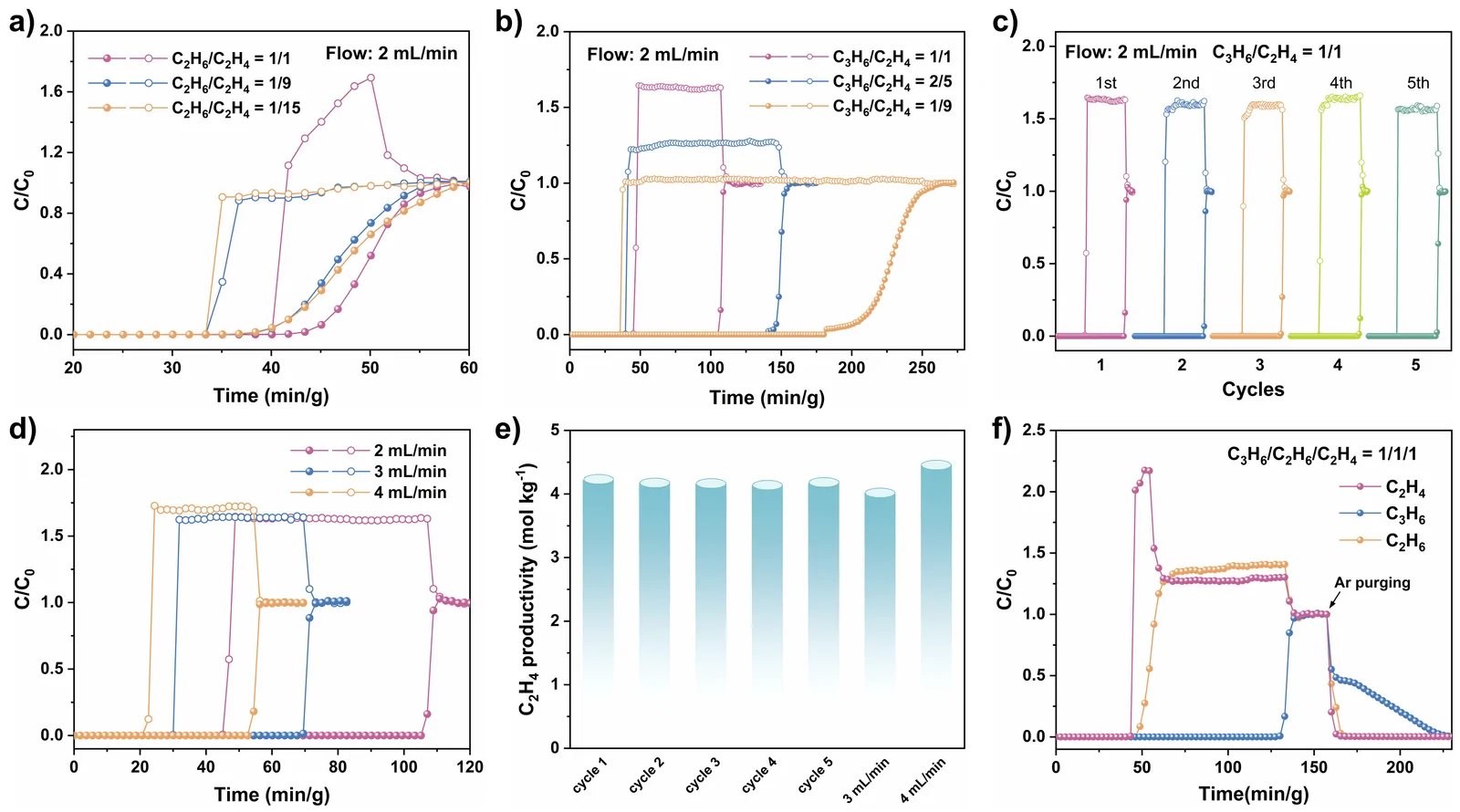
(a) Breakthrough curves for C_2_H_6_/C_2_H_4_ (1/1, 1/9 and 1/15, v/v) mixtures at 298 K with a total flow rate of 2.0 mL min^−1^; (b) breakthrough curves for C_3_H_6_/C_2_H_4_ (1/1, 2/5 and 1/9, v/v) mixtures at 298 K with a total flow rate of 2.0 mL min^−1^; (c) cycling breakthrough experiments for C_3_H_6_/C_2_H_4_ mixtures (1/1, v/v) at 298 K with a total flow rate of 2.0 mL min^−1^; (d) breakthrough curves for C_3_H_6_/C_2_H_4_ mixtures (1/1, v/v) at 298 K at different flow rates; (e) productivities of high-purity C_2_H_4_ under different working conditions. (f) Breakthrough curves for the C_3_H_6_/C_2_H_6_/C_2_H_4_ (1/1/1, v/v/v) mixtures at 298 K, after adsorption saturation, the desorption curves obtained under Ar (6 mL min^−1^) sweeping at 298 K.

Similar breakthrough experiments were conducted for C_3_H_6_/C_2_H_4_ mixtures at different ratios. As shown in Fig. 3b and S25–S27, a considerable amount of high-purity C_2_H_4_ (≥99.95%) was recovered before C_3_H_6_ broke through the column. Based on the breakthrough curves, the productivities of ≥99.95% pure C_2_H_4_ were estimated to be 94.45, 176.55, and 263.68 L(STP) kg^−1^ for 1/1, 2/5, and 1/9 C_3_H_6_/C_2_H_4_ mixtures, respectively (Table S8). Notably, the C_2_H_4_ productivity of 94.45 L kg^−1^ for the equimolar mixture surpasses that of several benchmark adsorbents, including FJI-H8-Me (85.8 L kg^−1^),^55^ JNU-74a (68.5 L kg^−1^)^56^ and SIFSIX-Cu-TPA (78.4 L kg^−1^)^57^ (Fig. S28).

Given that C_3_H_6_ is also a valuable product in MTO streams, desorption studies were performed under Ar flow (6 mL min^−1^) at 298 K after adsorption equilibrium (Fig. S25–S27). Specifically, when Ar was switched into the packed column, a small amount of C_2_H_4_ was desorbed quickly, and then high-purity C_3_H_6_ (≥99.5%) could be collected for a certain period of time. The recoverable amounts of C_3_H_6_ (≥99.5%) were estimated as 77.62, 66.10 and 33.84 L kg^−1^ from 1/1, 2/5, and 1/9 C_3_H_6_/C_2_H_4_ mixtures, respectively. NKU-400 maintains consistent separation performance over five consecutive adsorption–desorption cycles (Fig. 3c). In addition, the influence of gas flow rate on separation efficiency was investigated, and results indicated minimal impact, confirming the robustness of NKU-400 under varied operational conditions (Fig. 3d and e). The influence of humidity on dynamic separation was further evaluated using an equimolar C_3_H_6_/C_2_H_4_ mixture at 298 K under RH values of 33% and 53% (Fig. S29). The breakthrough profiles under humid conditions closely overlap with the profile obtained under dry condition, with C_2_H_4_ consistently eluting first and C_3_H_6_ remaining preferentially retained. The nearly unchanged elution sequence and separation window demonstrate that moderate humidity has a negligible influence on the dynamic C_3_H_6_/C_2_H_4_ separation performance of NKU-400.

Encouraged by its outstanding separation performance for binary C_3_H_6_/C_2_H_4_ and C_2_H_6_/C_2_H_4_ mixtures, NKU-400 was further assessed for the recovery of C_2_H_4_ and C_3_H_6_ from ternary C_3_H_6_/C_2_H_6_/C_2_H_4_ mixtures. As demonstrated in Fig. 3f and S30, the elution order followed C_2_H_4_, C_2_H_6_, and C_3_H_6_, consistent with their relative adsorption affinities. High-purity C_2_H_4_ (≥99.95%) was obtained prior to the breakthrough of C_2_H_6_ and C_3_H_6_. After adsorption saturation, the column was regenerated by Ar purging (6 mL min^−1^) at 298 K. This mild laboratory-scale condition was selected to promote desorption by lowering the partial pressures of the adsorbed hydrocarbons without introducing competitive adsorbates or additional thermal input. Under these conditions, C_2_H_4_ and C_2_H_6_ were preferentially removed, enabling the subsequent recovery of high-purity C_3_H_6_ (≥99.5%). Specifically, for the 1/1/1 and 10/2/25 C_3_H_6_/C_2_H_6_/C_2_H_4_ mixtures (v/v/v), the productivities of high-purity C_2_H_4_ were 1.83 L kg^−1^ and 0.51 L kg^−1^, respectively, while the corresponding recoverable amounts of high-purity C_3_H_6_ were 59.72 L kg^−1^ and 54.07 L kg^−1^, demonstrating the sequential recovery of both valuable olefins within a single adsorption–desorption cycle. Importantly, the ternary separation should not be interpreted as arising from equally high equilibrium selectivity for both the C_3_H_6_/C_2_H_4_ and C_2_H_6_/C_2_H_4_ pairs. Rather, the pronounced C_3_H_6_/C_2_H_4_ discrimination, together with the more modest C_2_H_6_/C_2_H_4_ discrimination, gives rise to the observed dynamic adsorption hierarchy and enables the stepwise recovery of polymer-grade C_2_H_4_ and high-purity C_3_H_6_ within a single adsorption–desorption cycle.

To further evaluate the influence of regeneration temperature, an additional desorption experiment was conducted for the equimolar C_3_H_6_/C_2_H_6_/C_2_H_4_ mixture at 353 K under the same Ar flow rate of 6 mL min^−1^ (Fig. S31). Increasing the temperature markedly accelerated the desorption process, particularly the release of the more strongly retained C_3_H_6_. However, the faster desorption also narrowed the time window over which ≥99.5% C_3_H_6_ could be selectively collected, resulting in a decrease in its high-purity productivity from 59.72 L kg^−1^ at 298 K to 35.82 L kg^−1^ at 353 K. These results reveal a trade-off between regeneration rate and selective product recovery: moderate heating facilitates more rapid desorption, whereas room-temperature Ar purging provides a more favorable temporal separation for the recovery of high-purity C_3_H_6_. The structural integrity of NKU-400 after multiple breakthrough experiments was confirmed by PXRD analysis (Fig. S32).

### 2.5 Mechanistic analysis

To gain a deeper understanding of the preferential adsorption of C_3_H_6_ and the distinct separation behaviors among C_3_H_6_, C_2_H_6_, and C_2_H_4_, grand canonical Monte Carlo (GCMC) simulations combined with dispersion-corrected density functional theory (DFT-D) calculations were performed. The GCMC simulations were first employed to identify the preferential adsorption sites of the three hydrocarbons in NKU-400, while subsequent DFT-D calculations were used to further optimize the host–guest configurations and evaluate their binding energies (*E*_b_). As illustrated in Fig. 4a–c and S33, the simulated density distributions and optimized binding geometries reveal that the primary trapping sites for all three hydrocarbons are located within the confined diamond-shaped channels, specifically nestled against the pore walls formed by two Zr_6_ clusters and three TCPPDA^4−^ ligands. Although the three gas molecules occupy similar adsorption domains, C_3_H_6_ exhibits a tighter fit and closer contact distances with the framework compared with C_2_H_6_ and C_2_H_4_, leading to the most favorable thermodynamic stabilization. The calculated binding energies are −37.64, −28.96 and −28.00 kJ mol^−1^ for C_3_H_6_, C_2_H_6_ and C_2_H_4_, respectively. The substantially more favorable binding energy of C_3_H_6_ is consistent with its distinctly higher experimental zero-coverage *Q*_st_ (29.65 kJ mol^−1^). By contrast, the calculated binding energies of C_2_H_6_ and C_2_H_4_ differ by only 0.96 kJ mol^−1^, consistent with their nearly identical zero-coverage *Q*_st_ values (23.78 and 23.74 kJ mol^−1^, respectively). Thus, the calculations primarily support the pronounced preference for C_3_H_6_, while the relatively modest C_2_H_6_/C_2_H_4_ discrimination should not be overinterpreted on the basis of individual binding energies alone.

**Fig. 4:**
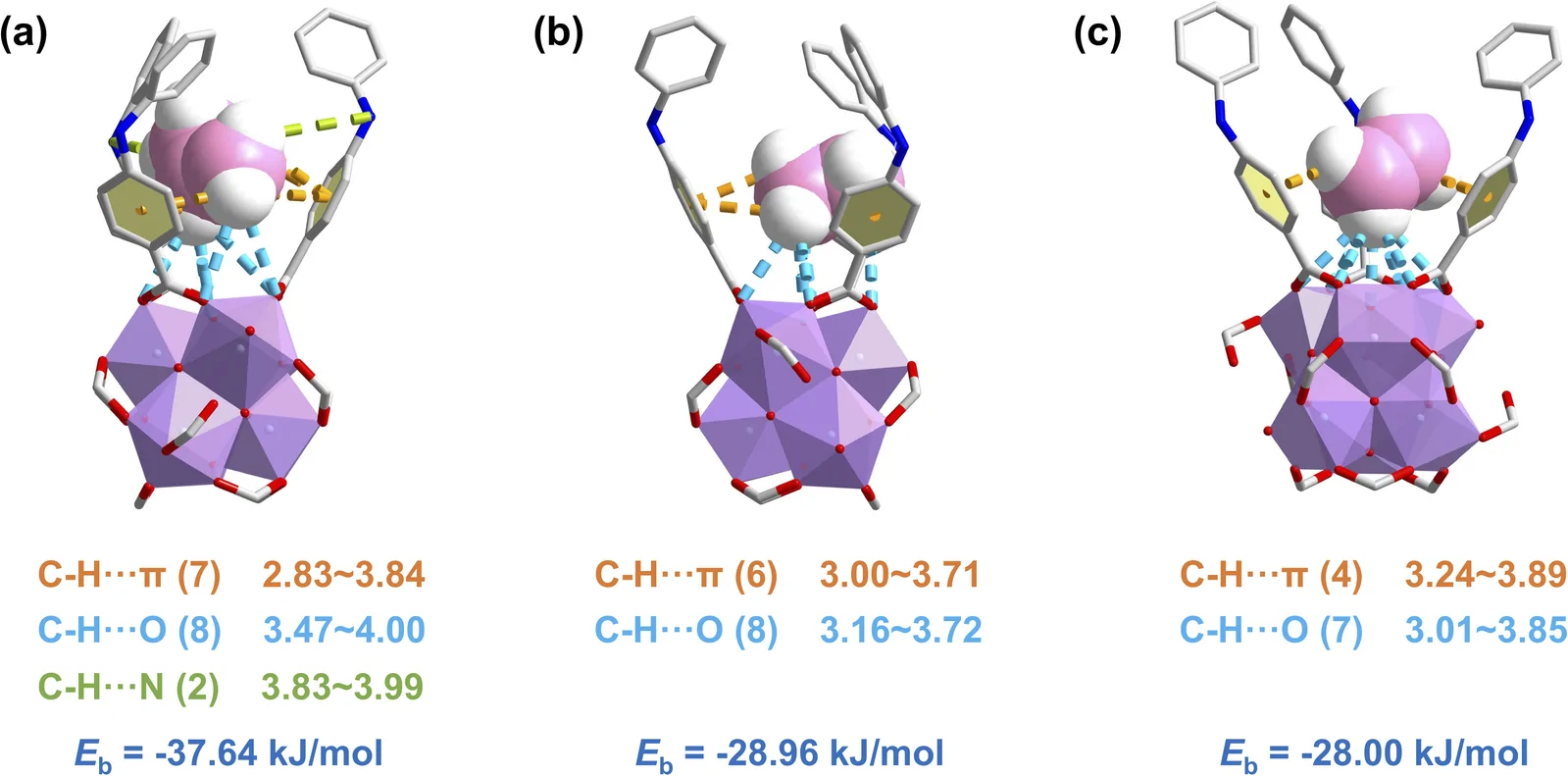
Preferential adsorption sites and interactions of (a) C_3_H_6_, (b) C_2_H_6_, and (c) C_2_H_4_ in NKU-400. All interatomic distances are given in Å. The orange, blue and green dashed lines represent C–H⋯π, C–H⋯O, and C–H⋯N interactions, respectively.

Specifically, each C_3_H_6_ molecule is anchored by an intricate network of multiple weak interactions. It interacts with three neighboring phenyl rings through seven C–H⋯π contacts (*d*_H⋯π_ = 2.83–3.84 Å) and is further stabilized by eight C–H⋯O contacts (*d*_H⋯O_ = 3.47–4.00 Å) and two C–H⋯N contacts (*d*_H⋯N_ = 3.83–3.99 Å) (Fig. 4a). The high density of cooperative interactions, especially the more pronounced involvement of the aromatic π-rich pore surface, accounts for the significantly stronger binding affinity of C_3_H_6_. In the case of C_2_H_6_, although it lacks the π-electrons of an alkene, its relatively larger molecular size and higher polarizability compared with C_2_H_4_ enable more extensive van der Waals contacts with the pore surface. C_2_H_6_ establishes six C–H⋯π interactions (*d*_H⋯π_ = 3.00–3.71 Å) and eight C–H⋯O interactions (*d*_H⋯O_ = 3.16–3.72 Å), resulting in a moderate binding affinity (Fig. 4b). By contrast, C_2_H_4_ possesses the smallest molecular dimensions and fits more loosely within the channels of NKU-400. This less favorable spatial matching reduces the number and strength of host–guest contacts, involving only four C–H⋯π contacts (*d*_H⋯π_ = 3.24–3.89 Å) and seven C–H⋯O contacts (*d*_H⋯O_ = 3.01–3.85 Å) (Fig. 4c).

Overall, the combined GCMC simulations and DFT-D calculations demonstrate that the adsorption preference follows the sequence C_3_H_6_ > C_2_H_6_ > C_2_H_4_, which arises from the synergistic effect of steric confinement, molecular polarizability, and multiple host–guest interactions. The aromatic-rich and N/O-functionalized pore surfaces of NKU-400 provide a tailor-made confined environment that preferentially stabilizes larger and more polarizable hydrocarbons, especially C_3_H_6_. Meanwhile, the slightly stronger interaction with C_2_H_6_ than with C_2_H_4_ is consistent with their close but distinguishable adsorption affinities, thereby contributing to the efficient one-step purification of C_2_H_4_ from complex MTO product mixtures.

## 3 Conclusions

In summary, we developed a highly robust zirconium-based MOF, NKU-400, featuring a unique (4,4,12)-connected topology, uniform one-dimensional channels, and aromatic-rich pore surfaces decorated with accessible N/O sites. The tailored pore environment affords a pronounced thermodynamic preference for C_3_H_6_ over C_2_H_4_, while the more modest C_2_H_6_/C_2_H_4_ discrimination develops at finite loading. GCMC and DFT calculations reveal that pore confinement and multiple C–H⋯π/O/N interactions cooperatively stabilize C_3_H_6_ within the framework. As a result, NKU-400 combines high C_3_H_6_ uptake with high C_3_H_6_/C_2_H_4_ selectivity and moderate adsorption enthalpies. Dynamic breakthrough experiments deliver polymer-grade C_2_H_4_ (≥99.95%) with a productivity of 94.45 L(STP) kg^−1^ from an equimolar C_3_H_6_/C_2_H_4_ mixture, followed by the recovery of high-purity C_3_H_6_ (≥99.5%). NKU-400 further enables the one-step purification of polymer-grade C_2_H_4_ from ternary C_3_H_6_/C_2_H_6_/C_2_H_4_ mixtures. Notably, its dynamic C_3_H_6_/C_2_H_4_ separation performance is essentially maintained under moderate humid conditions (RH = 33% and 53%). Together with its excellent chemical robustness, these results highlight NKU-400 as a promising adsorbent for MTO-derived light-hydrocarbon purification and demonstrate the effectiveness of integrating topology design with pore-surface engineering for challenging multicomponent separations.

## Author contributions

R. Zheng, J. Xu and X.-H. Bu conceived and designed the experiments. R. Zheng conducted the experiments, performed the data analysis, and drafted the manuscript; Q. Zhang, L.-L. Wang, M.-W. Wan and Z.-Q. Yao contributed to the synthesis and some characterization experiments. T.-L. Hu, J. Xu and X.-H. Bu reviewed and revised this manuscript.

## Conflicts of interest

There are no conflicts to declare.

## Supplementary Material

SC-OLF-D6SC05534F-s001

## Data Availability

CCDC 2527572 (NKU-400) contains the supplementary crystallographic data for this paper.^58^ The data supporting this article are included in the main text and the supplementary information (SI). Supplementary information: experimental procedures, characterization data, gas adsorption measurements, IAST calculations, dynamic breakthrough data, and computational details. See DOI: https://doi.org/10.1039/d6sc05534f.
