# Supplementary material for: Topology and pore-surface engineering of a Zr-MOF for ethylene purification and propylene recovery from MTO products
Source: Chem Sci. 2026 Oct 1. Online ahead of print. doi: 10.1039/d6sc05534f (PMC13643523; doi:10.1039/d6sc05534f)
Supplement: Supplementary file 1 [file SC-OLF-D6SC05534F-s001.pdf]

Supplementary Information (SI) for Chemical Science.
This journal is © The Royal Society of Chemistry

Supporting Information

Topology and Pore-Surface Engineering of a Zr-MOF for Ethylene Purification and Propylene Recovery from MTO Products

Ran Zheng,[a] Qiang Zhang,[a] Lulu Wang,[a] Mingwei Wan,[a] Zhaoquan Yao,[ab] Tongliang Hu,[a] Jian Xu*[a] and Xian-He Bu[ac]

[a] *School of Materials Science and Engineering, Smart Sensing Interdisciplinary Science Center, TKL of Metal and Molecule-Based Material Chemistry, Nankai University, Tianjin 300350, China.*

[b] *School of Chemistry and Chemical Engineering, Tianjin University of Technology, Tianjin, 300384, China.*

[c] *State Key Laboratory of Elemento-Organic Chemistry, Frontiers Science Center for New Organic Matter, Collaborative Innovation Center of Chemical Science and Engineering (Tianjin), Nankai University, Tianjin 300071, China.*

* *Corresponding authors. E-mail address: jxu@nankai.edu.cn (J. Xu).*

Table of Contents

Section S1: Materials and instrumentation

Section S2: Synthesis of NKU-400

Section S3: Single-crystal X-ray diffraction analysis (SCXRD)

Section S4: Additional Structural Information

Section S5: Structure Characterization

Section S6: Gas adsorption measurements and analysis

Section S7: Dynamic breakthrough experiments

Section S8: Theoretical method

References

Section S1: Materials and instrumentation

Chemicals and reagents: All chemicals and reagents were received from commercial suppliers and were used directly without additional purification. $ZrCl_4$ (purity ≥ 98%) was purchased from J&K Scientific Co., Ltd. N,N,N',N'-tetrakis(4-carboxyphenyl)-1,4-phenylenediamine (H_4TCPPDA, purity ≥ 95%) was purchased from Shanghai Bide Pharmaceutical Technology Co., Ltd. N,N-dimethylformamide (DMF, GR, purity ≥ 99.5%) was purchased from Concord Technology Co., Ltd. Benzoic acid (BA, purity ≥ 99%) was purchased from Aladdin Industrial Inc (Shanghai, China).

Single-crystal X-ray diffraction (SCXRD): Single-crystal X-ray diffraction data were collected with the Rigaku XtaLAB PRO MM007 DW diffractometer using Cu-Kα radiation (λ = 1.5418 Å) at 100 K. The structure was solved by *SHELXT* program and refined by full-matrix least-squares against F^2 using the *SHELXL* program [1-3]. Anisotropic thermal parameters were implemented to all non-hydrogen atoms, and all hydrogen atoms were placed in the calculated positions and refined with isotropic thermal parameters. Details of the crystal parameters, data collection, and refinement are listed in Table S1.

Powder X-ray diffraction (PXRD): PXRD patterns were collected on a Rigaku MiniFlex 600 diffractometer equipped with a Cu-target tube at 40 kV and 15 mA over the range from 3° to 50°. The samples were placed on zero-background silicon crystal plates before measurements.

Elemental Analyses (EA): EA measurements were performed on a Vario Elementar Cube elemental analyzer. Prior to EA measurements, the sample was thoroughly washed with acetone for 72 h and activated under dynamic vacuum at 150 °C for 12 h.

Thermogravimetric analysis (TGA): TGA was performed on a Rigaku standard TG-DTA analyzer in the range from room temperature to 800 °C with a heating rate of 10 °C min^{-1} under air atmosphere, using an empty Al_2O_3 crucible as the reference.

Gas adsorption Measurements: Adsorption–desorption isotherms of C_3H_6, C_2H_6, C_2H_4 and N_2 were measured using a Micromeritics ASAP 2460 volumetric adsorption analyzer over a pressure range of 0–1.0 bar. More than 100 mg of the sample was used

for adsorption studies. Before measurements, the solvent-exchanged sample was degassed at 150 °C under high vacuum for 12 h.

Water vapor adsorption measurements: Water vapor adsorption–desorption isotherms were obtained on a BELSORP MAX II instrument at 298 K. The measurements were conducted across a relative humidity (RH) range of 2.5% – 95%, with a maximum equilibrium time of 300 s allowed for each RH step. Before the measurement, the solvent-exchanged sample was degassed at 150 °C under high vacuum for 12 h.

Breakthrough Experiments: Dynamic breakthrough tests were performed using a custom-built separation apparatus. Approximately 0.5 g of activated NKU-400 sample was packed into a stainless-steel column with an internal diameter of 3.0 mm and a length of 200 mm. Prior to the test, the column was purged with a He flow (40 mL min^{-1}) for 120 minutes. The composition of the effluent gas mixture was continuously monitored using an online gas chromatograph (GC-7800). All gas volumes and volumetric productivities derived from the breakthrough experiments are reported at STP (273.15 K and 1 atm). All gases were obtained from commercial companies.

Section S2: Synthesis of NKU-400

A mixture of $ZrCl_4$ (0.9 mmol, 210 mg), $H_4TCPPDA$ (0.12 mmol, 70 mg), benzoic acid (7.0 g), and N,N- dimethylformamide (DMF, 14 mL) was ultrasonically dissolved in a 20 mL scintillation vial. The vial was sealed and heated at 140 °C for 48 hours under solvothermal conditions. After cooling to room temperature, the light-yellow crystals were collected by centrifugation, sequentially washed with DMF, and dried in the air, affording a yield of 60.1% (based on $H_4TCPPDA$). Elemental analysis (%): calcd: C 49.53, H 2.91, N 3.40; found: C 49.76, H 2.73, N 3.36.

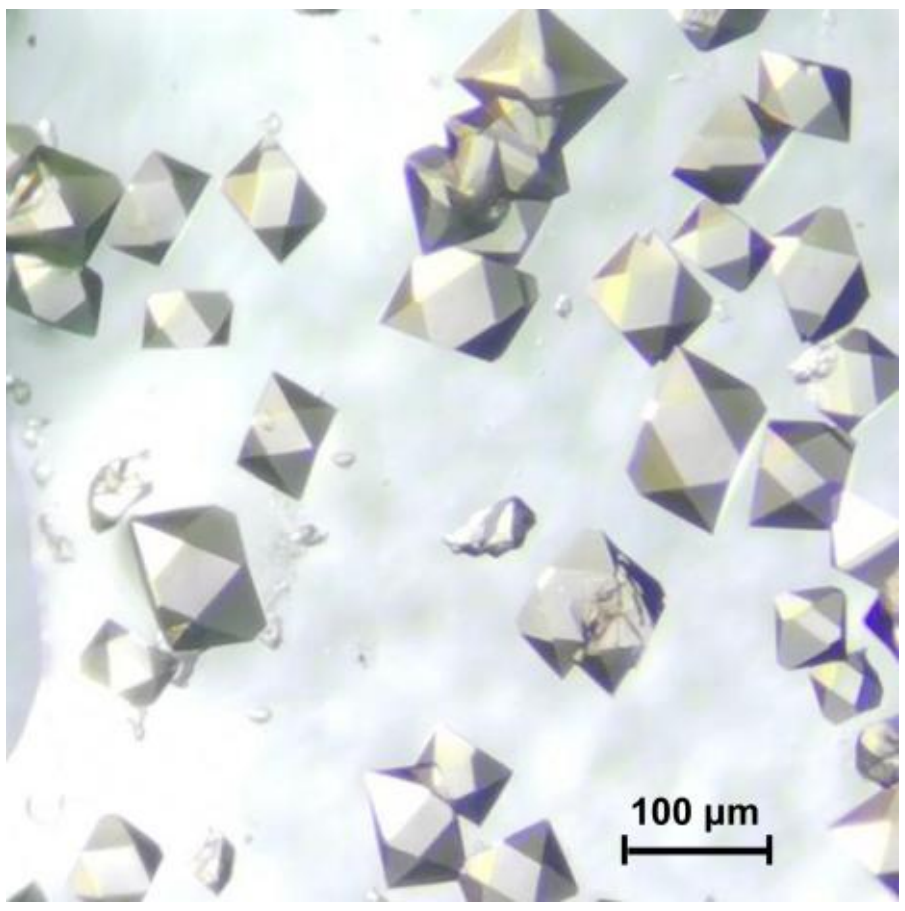

Fig. S1 Optical image of the single crystal of NKU-400.

Section S3: Single-crystal X-ray diffraction analysis (SCXRD)

Table S1. Crystallographic data and structure refinement results for NKU-400.

Compound	NKU-400
Chemical formula	$C_{51}H_{36}N_3O_{17}Zr_3$
CCDC number	2527572
Formula weight	1236.49
T/K	99.99
Crystal system	monoclinic
Space group	*C*2/*c*
a/Å	22.2447(2)
b/Å	27.3434(3)
c/Å	30.8176(2)
α/°	90
β/°	100.9800(10)
γ/°	90
Volume/Å^3	18401.5(3)
Z	8
ρ_{calc}/g cm^{-3}	0.893
μ/mm^{-1}	3.095
F(000)	4952.0
Radiation	Cu Kα (λ = 1.54184)
2Θ range for data collection/°	5.178 to 153.486
Index ranges	$-27 \le h \le 28$, $-29 \le k \le 34$, $-38 \le l \le 21$
Reflections collected	64138
Independent reflections	18584 [R_{int} = 0.0468, R_{sigma} = 0.0411]
Data/restraints/parameters	18584/0/667
Goodness-of-fit on F^2	1.065
Final R indexes [I >= 2σ(I)]	R_1 = 0.0669, wR_2 = 0.1760
Final R indexes [all data]	R_1 = 0.0722, wR_2 = 0.1808

$^aR_1 = \sum||F_o| - |F_c||/\sum|F_o|$; $^bwR_2 = \{\sum[w(F_o^2 - F_c^2)^2] / \sum w(F_o^2)^2\}^{1/2}$.

Section S4: Additional Structural Information

Table S2. Summary of coordination modes and dihedral angles of $H_4TCPPDA$ ligands in NKU-400, P: central phenyl ring; P1 and P2: peripheral phenyl rings; (color scheme: Zr, pale blue; O, red; N, blue; C, gray. Hydrogen atoms were omitted for clarity).

Coordination mode and conformation of $H_4TCPPDA$	Dihedral angles between			
	P-P1	P-P2	P-P3	P-P4
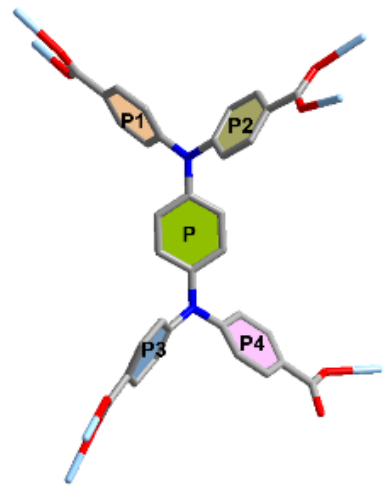 Type I	68.1°	66.4°	71.7°	60.7°
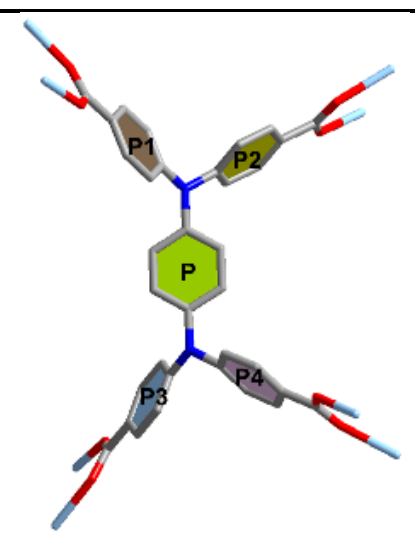 Type II	66.7°	76.3°	67.6°	76.9°

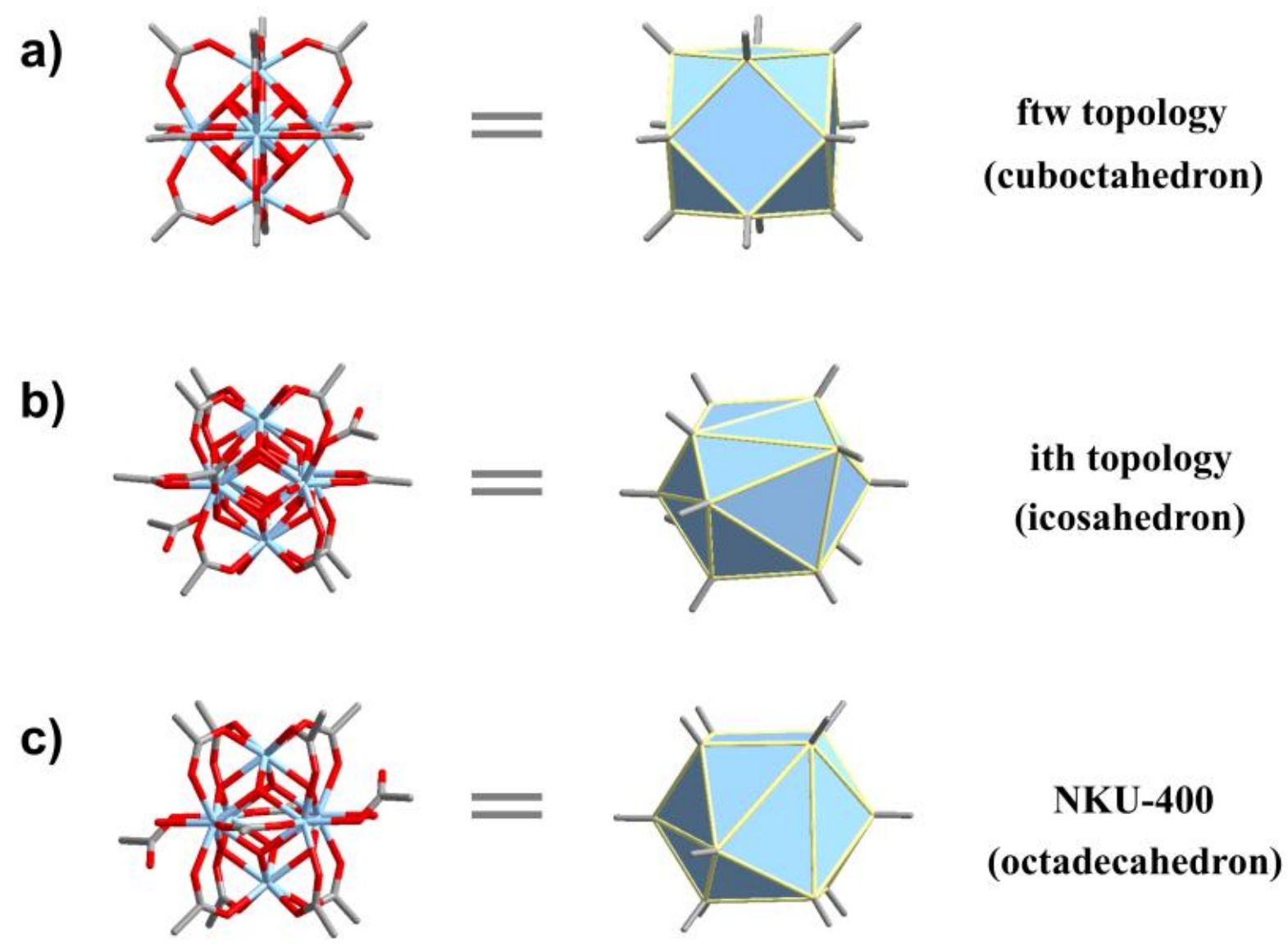

Fig. S2 Comparison of three typical 12-connected Zr_6 clusters.

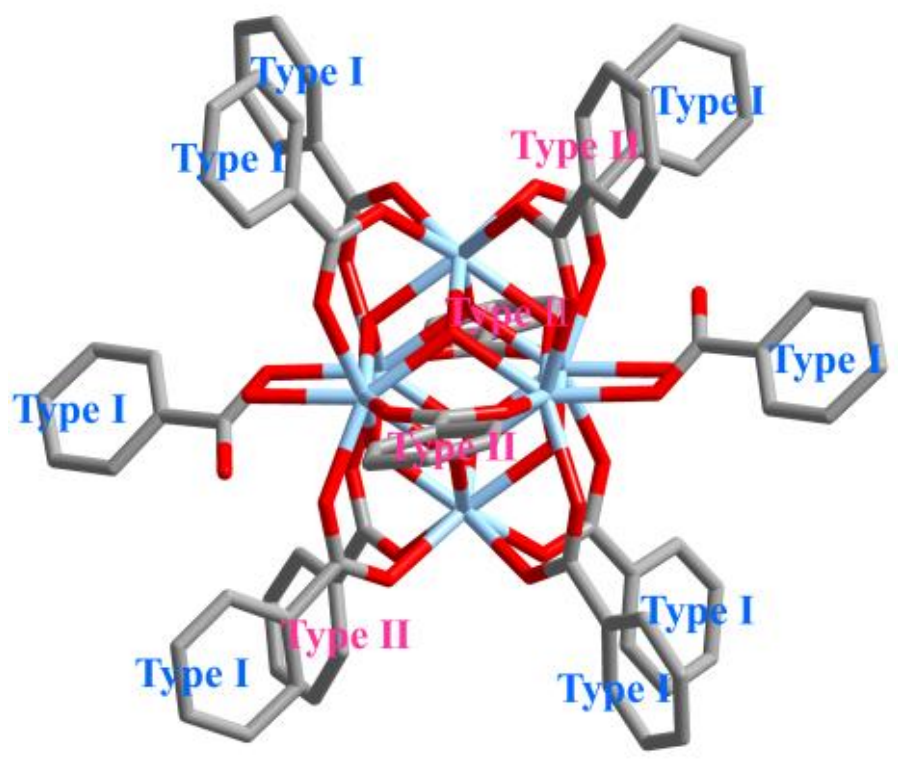

Fig. S3 The Zr_6 cluster in **NKU-400** forms a 12-connected coordination with eight type I ligands (blue) and four type II ligands (pink).

Section S5: Structural Characterization

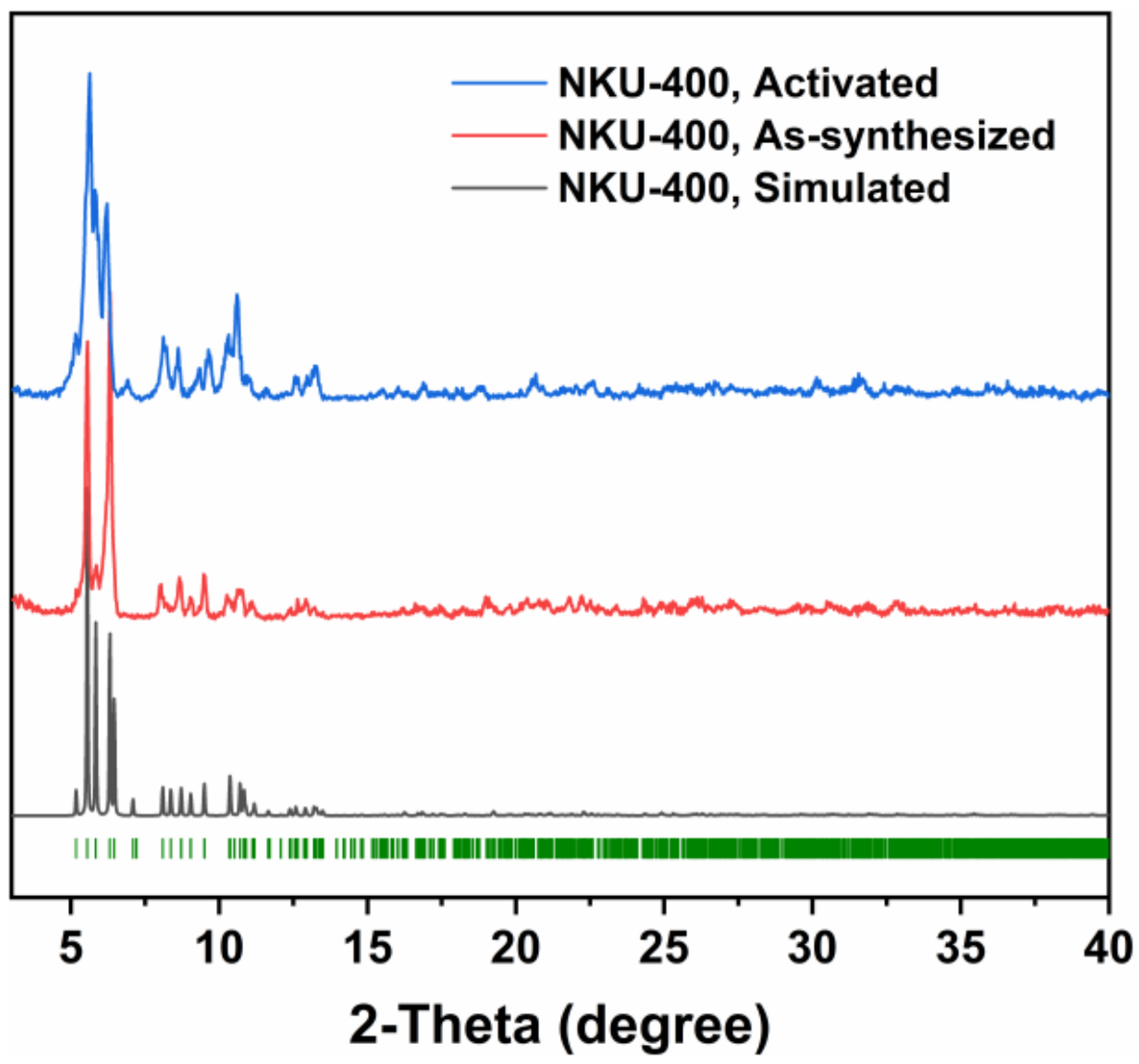

Fig. S4 Experimental and simulated PXRD patterns of NKU-400.

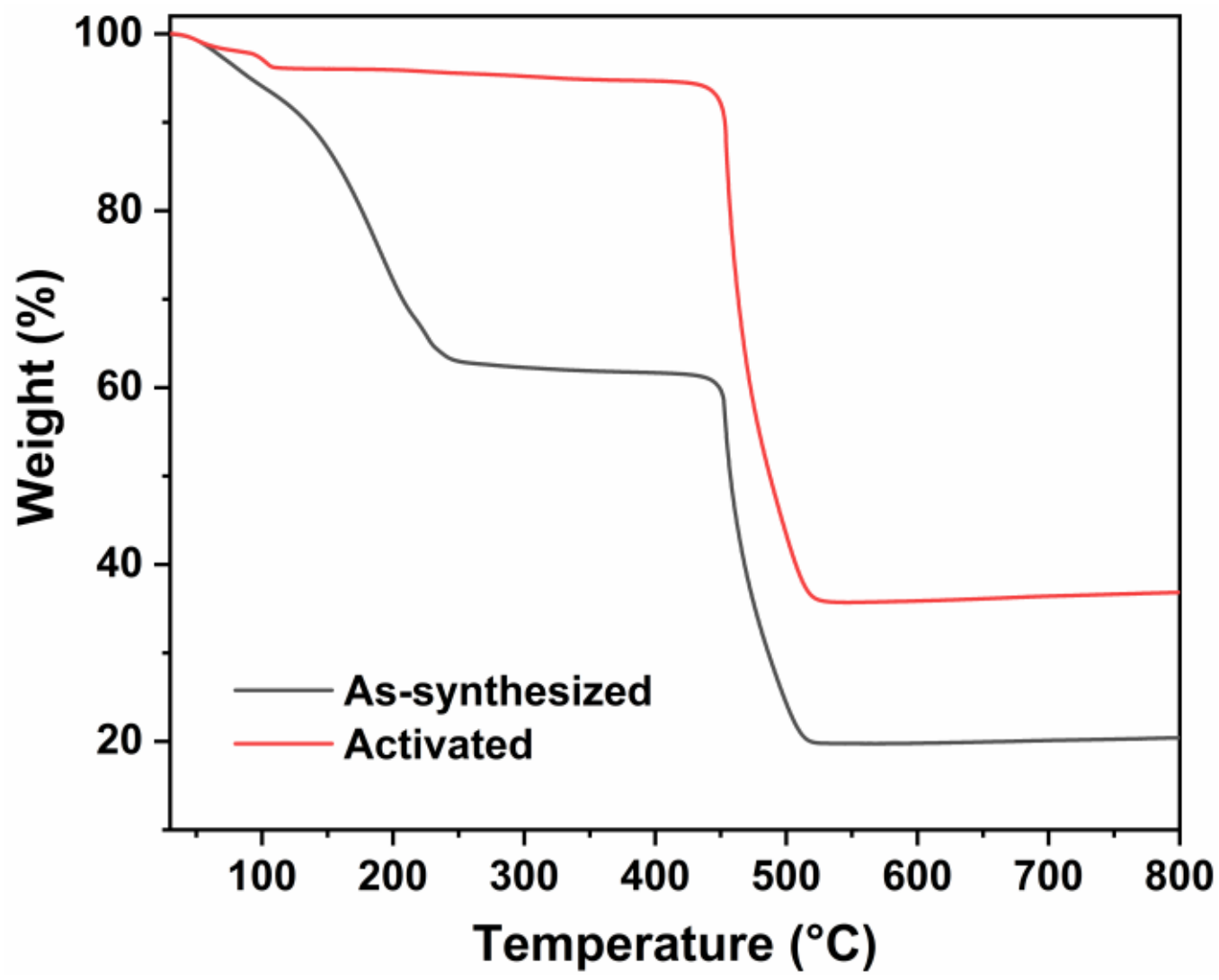

Fig. S5 TGA curves of the as-synthesized and activated samples of NKU-400.

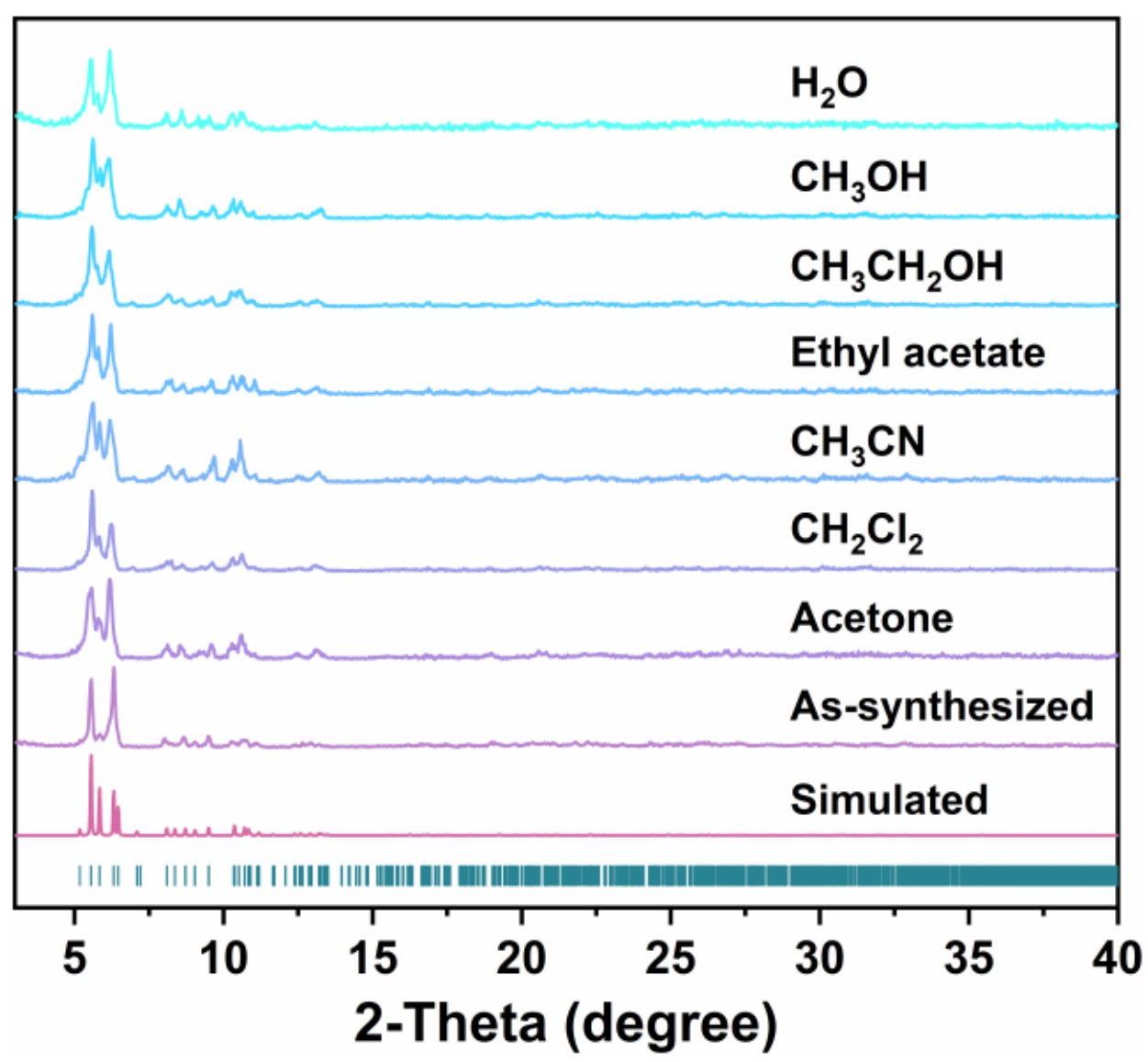

Fig. S6 PXRD patterns of NKU-400 in different solvents for 48 hours.

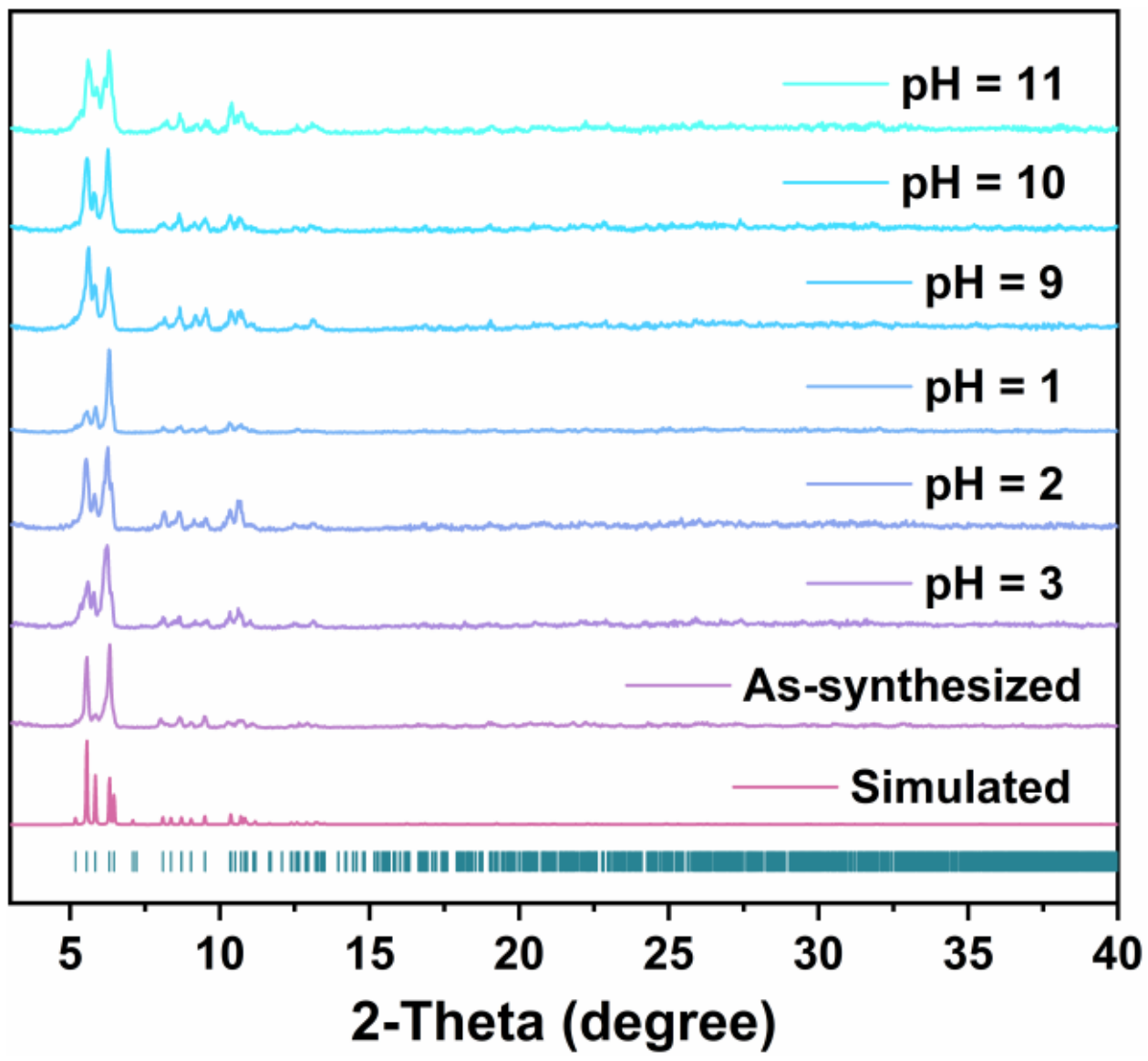

Fig. S7 PXRD patterns of NKU-400 in different pH solutions for 48 hours.

Section S6: Gas adsorption measurements and analysis

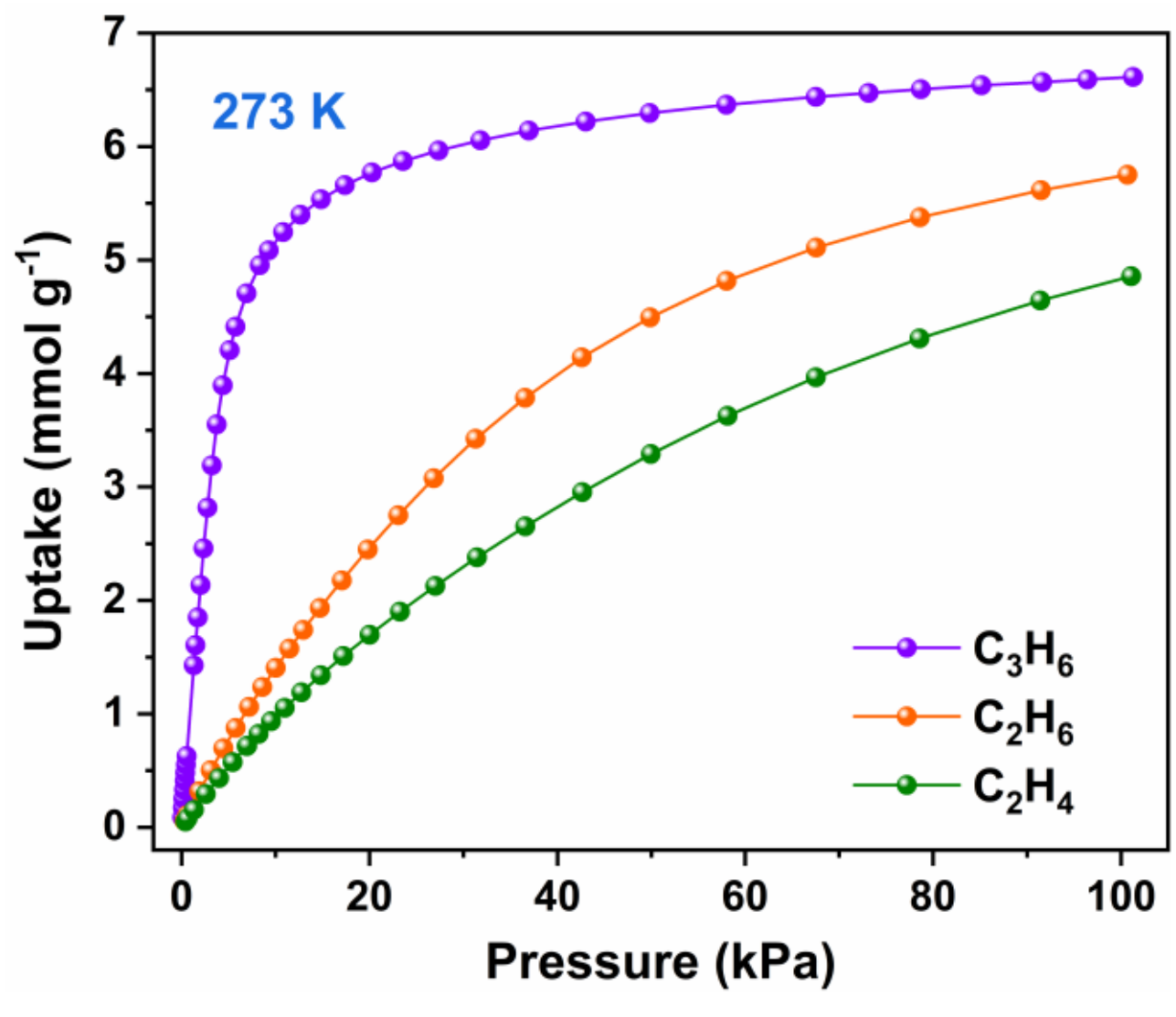

Fig. S8 The adsorption isotherms of NKU-400 for C_3H_6, C_2H_6 and C_2H_4 at 273 K.

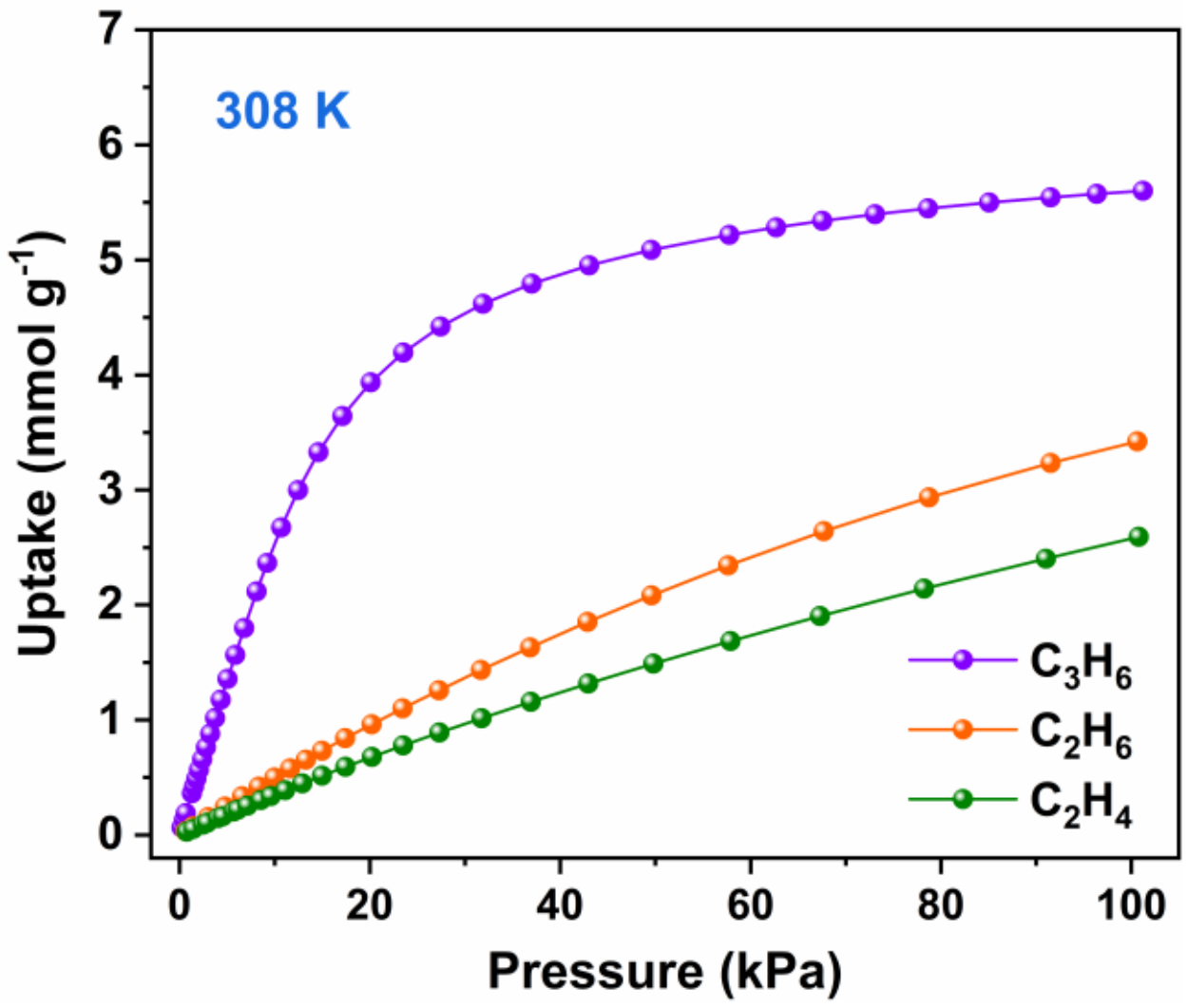

Fig. S9 The adsorption data of NKU-400 for C_3H_6, C_2H_6 and C_2H_4 at 308 K.

Table S3. Comparison of the gravimetric and volumetric C_3H_6 adsorption capacities of NKU-400 and other MOFs.

Adsorbent	C_3H_6 uptake (cm^3 g^{-1})	Crystal density (g cm^{-3})	Volumetric uptake (cm^3(STP) cm^{-3})	Ref.
NKU-400	134.55	0.893	120.15	**This work**
Zn-BPZ-SA	105.95	1.002	106.16	4
$Cu_3(OH)_2(Me_2BPZ)_2$	137.98	0.917	126.52	5
ZJNU-401	101.47	1.011	102.58	6
NUM-27a	53.83	1.756	94.53	7
HIAM-301	70.78	1.276	93.00	8
NTU-68	53.10	1.317	69.93	9
NCU-20	59.56	1.585	94.41	10
ZSTU-10	51.66	1.891	97.7	11
JNU-74a	94.53	1.219	115.23	12
spe-MOF	236.90	0.704	166.77	13
SIFSIX-Cu-TPA	128.90	1.078	138.95	14
Co-aip-pyz	37.91	1.678	63.62	15

Calculation of isosteric enthalpy of adsorption (Q_{st}) by Virial equation

The adsorption isotherms of C_3H_6, C_2H_6, and C_2H_4 at 273, 298 K and 308 K were fitted based on the Virial equation firstly[16], which can be written as:

$$\ln(P) = \ln(N) + \frac{1}{T}\sum_{i=0}^{m} a_i N^i + \sum_{j=0}^{n} b_j N^j \qquad (1)$$

where P is pressure (Pa), N denotes the amount adsorbed at pressure P (mmol g^{-1}), T is temperature (K), m and n refer to the number of terms required to adequately describe the isotherm, and a_i and b_j are empirical parameters.

Based on the virial coefficients obtained from the fitted isotherms, the isosteric heat of adsorption (Q_{st}) can be calculated by the equation:

$$Q_{st} = -R\times\sum_{i=0}^{m} a_i \times N^i \qquad (2)$$

Where R is a constant with the value of 8.314 J mol^{-1} K^{-1}.

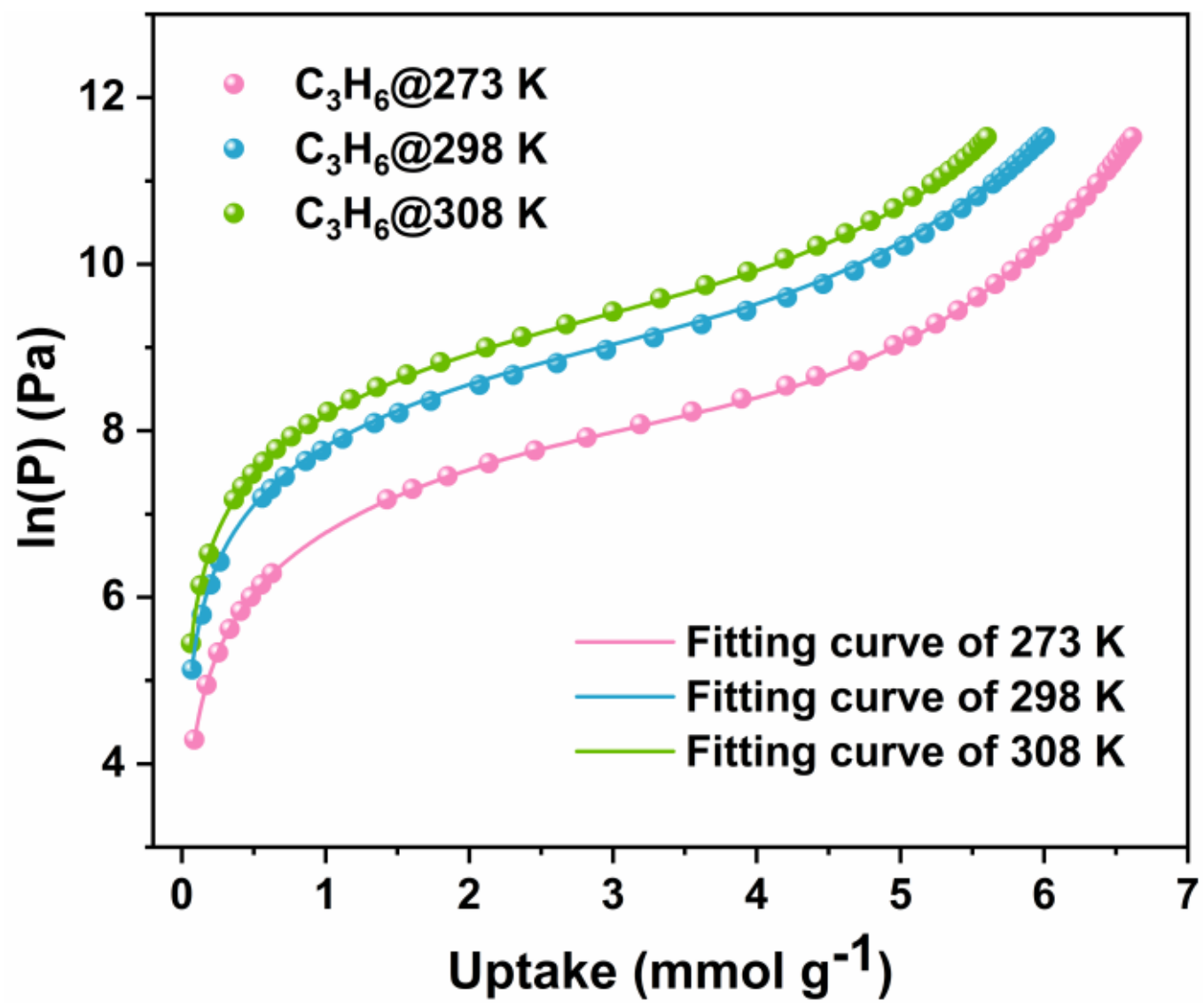

Fig. S10 Virial fitting of the C_3H_6 adsorption isotherms on NKU-400 measured at 273 K, 298 K and 308 K.

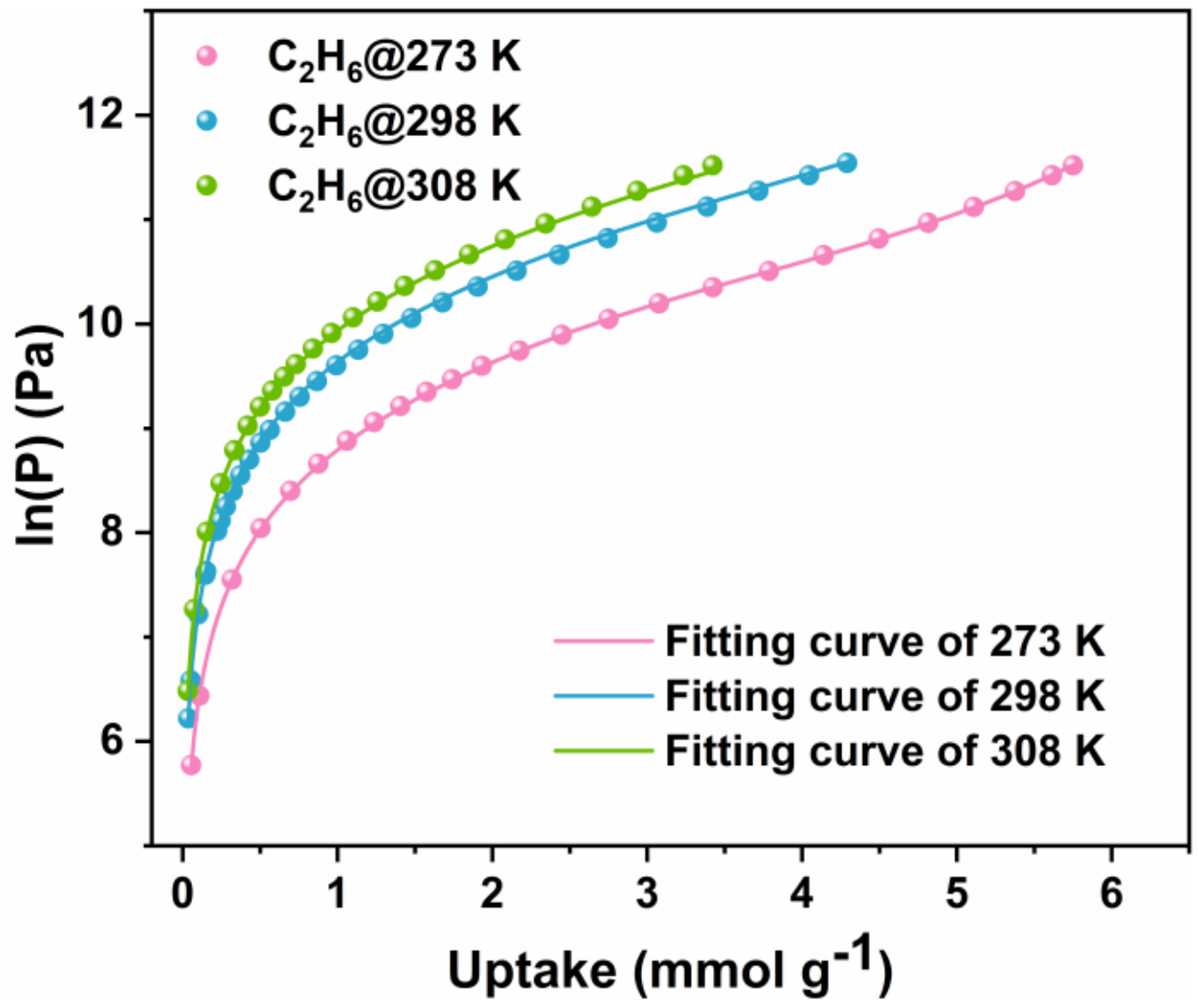

Fig. S11 Virial fitting of the C_2H_6 adsorption isotherms on NKU-400 measured at 273 K, 298 K and 308 K.

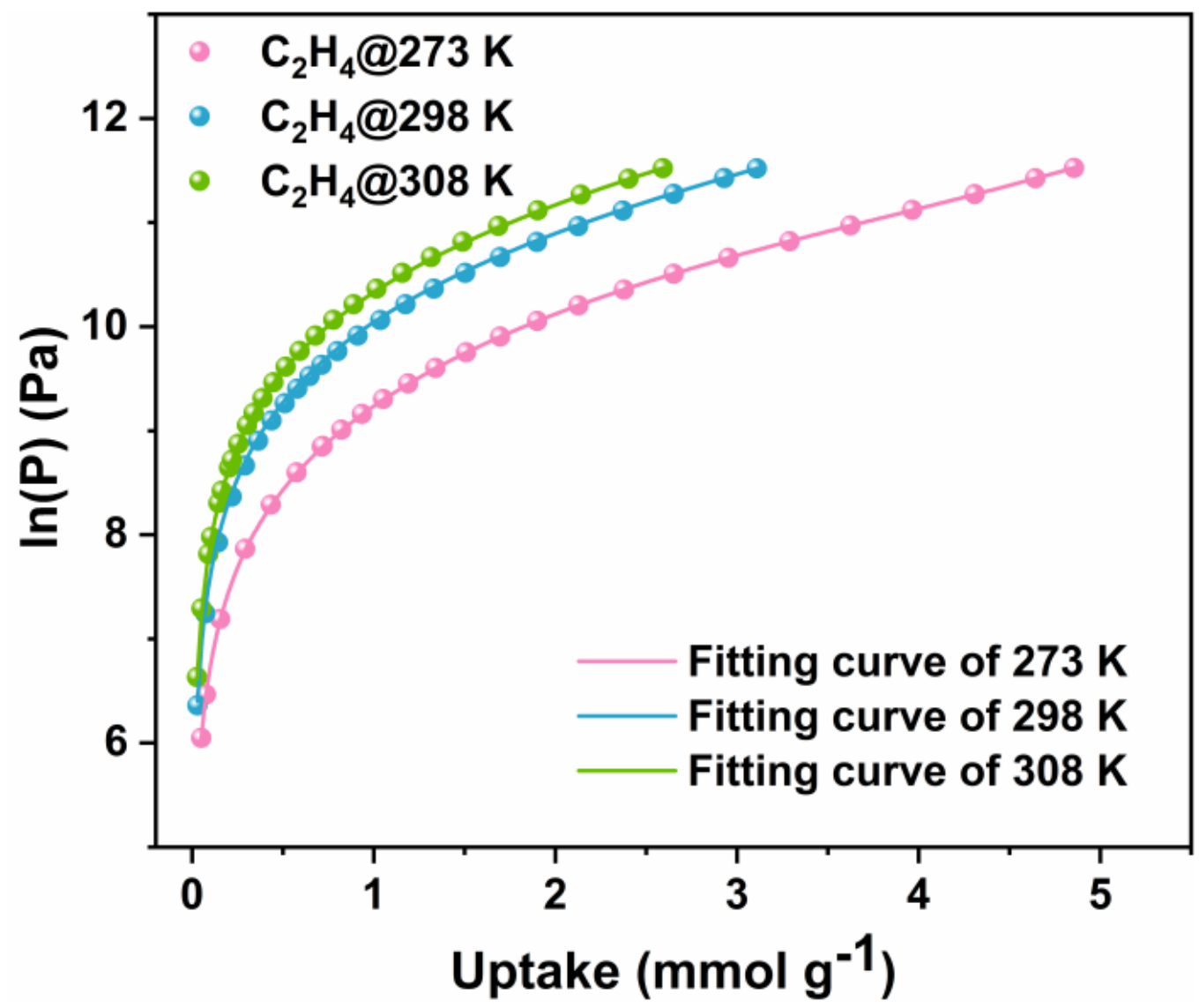

Fig. S12 Virial fitting of the C_2H_4 adsorption isotherms on NKU-400 measured at 273 K, 298 K and 308 K.

Table S4. The fitted parameters of the virial equation for NKU-400.

	C_3H_6	C_2H_6	C_2H_4
a_0	-3580.18644	-2865.68254	-2868.36371
a_1	266.88172	145.56739	303.03575
a_2	-59.0775	-18.06846	-59.94784
a_3	-8.40769	-4.3087	-2.75116
a_4	1.30119	0.5828	0.45132
b_0	19.81334	19.14384	19.5164
b_1	-0.93302	-0.40498	-0.87753
b_2	0.27175	0.09811	0.22138
Adj. R-Square	0.99994	0.99994	0.99997

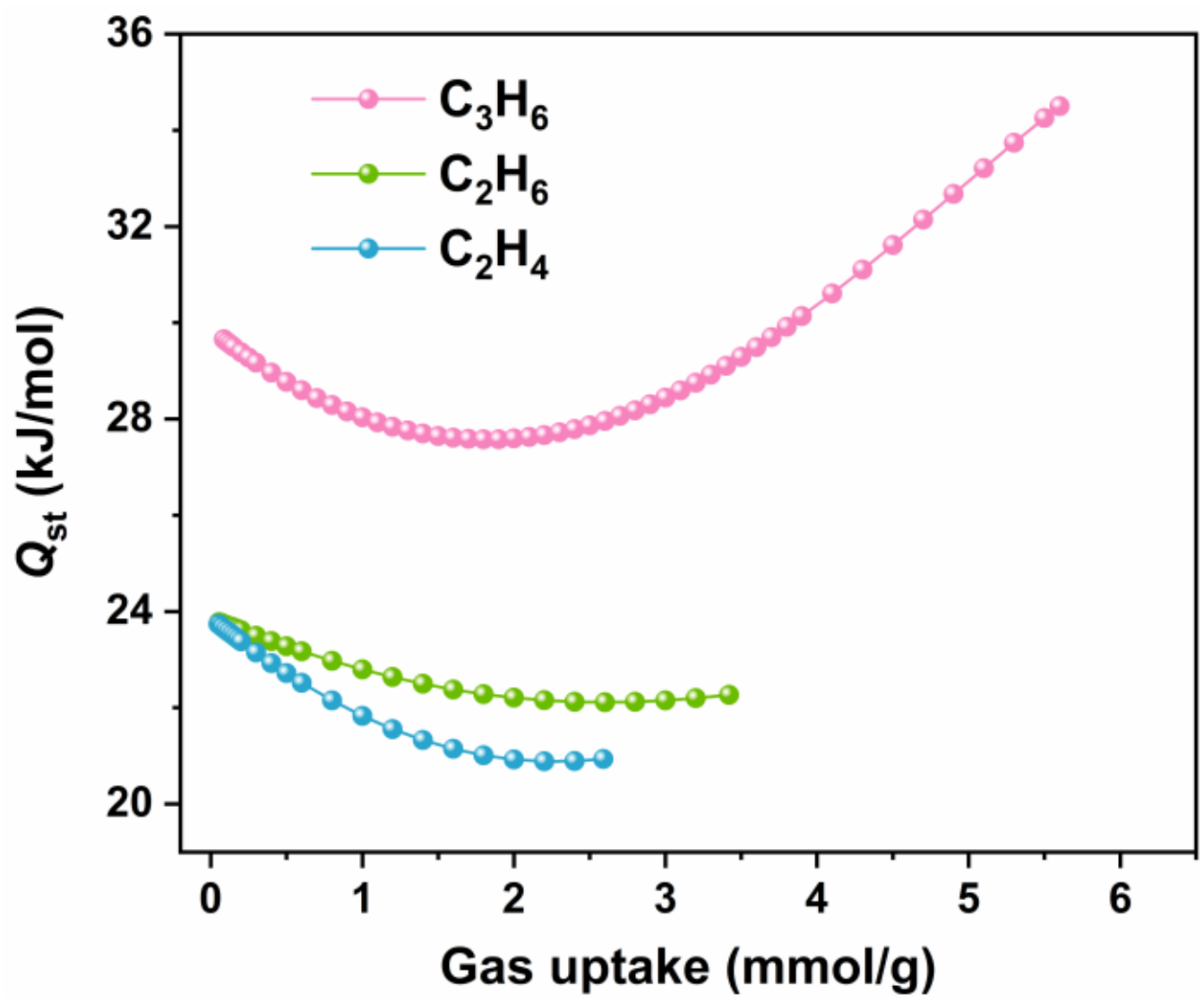

Fig. S13 Coverage-dependent isosteric heats of adsorption (Q_{st}) of C_3H_6, C_2H_6, and C_2H_4 in NKU-400 derived from the three-temperature Virial fitting.

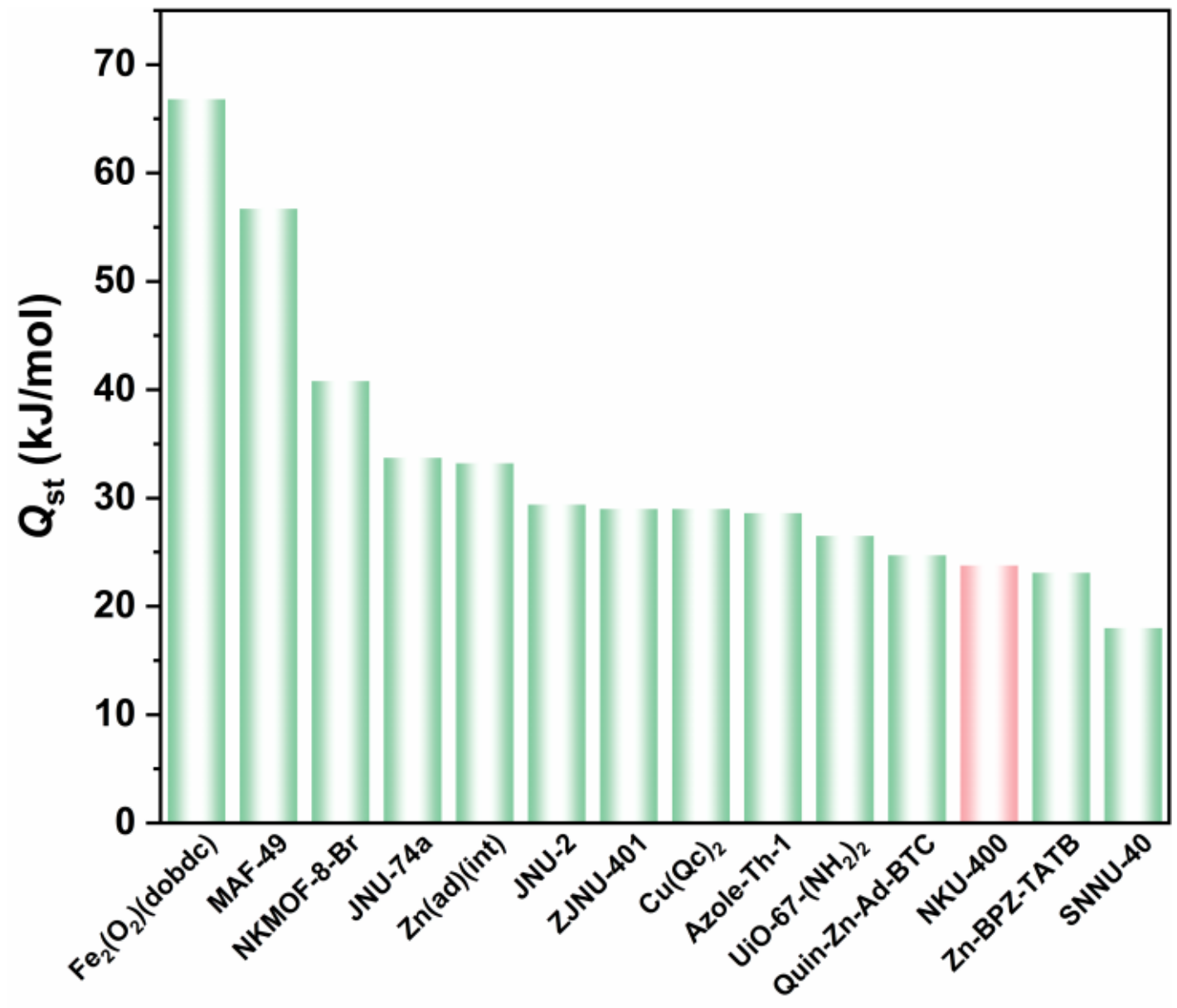

Fig. S14 Comparison of the zero-coverage Q_{st} of C_2H_6 among some representative C_2H_6-selective MOFs.

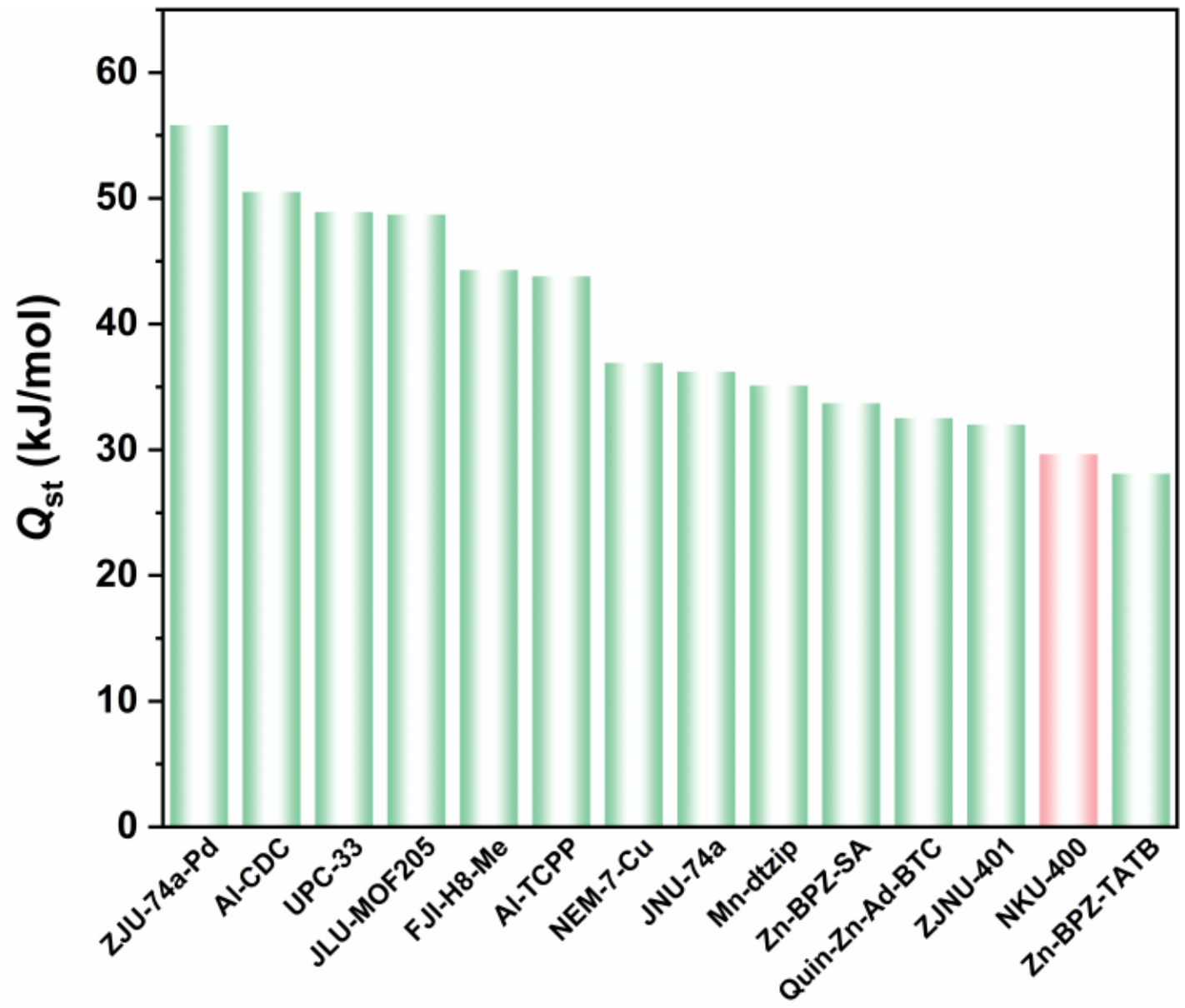

Fig. S15 Comparison of the zero-coverage Q_{st} of C_3H_6 among some representative C_3H_6-selective MOFs.

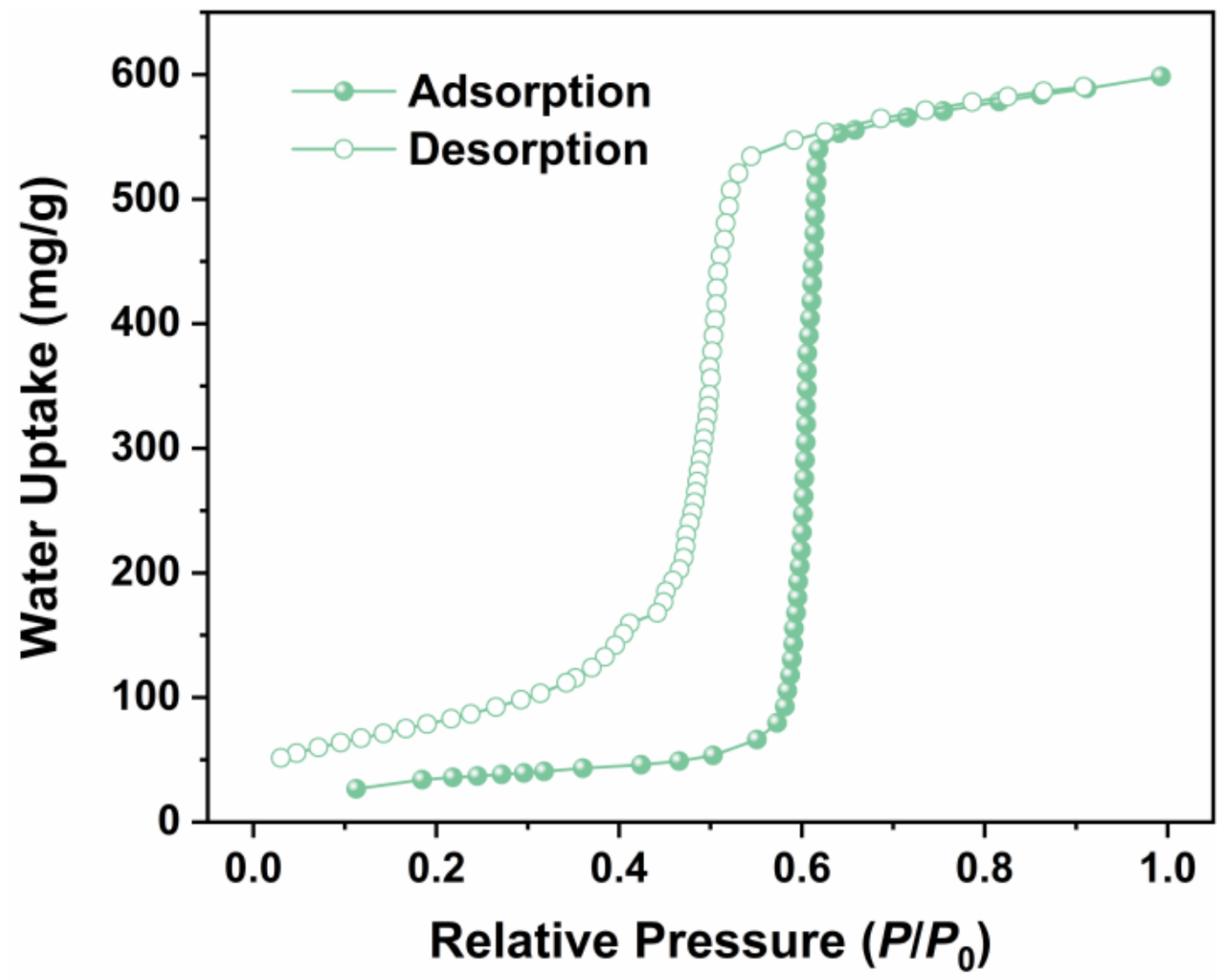

Fig. S16 The water vapor adsorption-desorption isotherm of NKU-400 at 298 K.

Calculation of IAST selectivity

The single-component adsorption isotherms of C_3H_6, C_2H_6, and C_2H_4 at 273 K and 298 K were fitted with Dual-sites Langmuir-Freundlich model (DSLF) mode (Equation 3).

$$q = q_A \frac{b_A p^{vA}}{1+b_A p^{vA}} + q_B \frac{b_B p^{vB}}{1+b_B p^{vB}} \qquad (3)$$

where p (kPa) is the pressure of the gas phase and the adsorbed phase at equilibrium, q is the adsorbed amount per mass of adsorbent (mmol g^{-1}), q_A and q_B are the saturation adsorption amount of site A and B (mmol g^{-1}), b_A and b_B are the affinity coefficients of site A and B (kPa), and v_A and v_B represent the deviations from an ideal homogeneous surface.

The adsorption selectivity was established by Ideal Adsorbed Solution Theory (IAST) [17], the selectivity has been defined as equation 4:

$$S = \frac{q_1 p_2}{q_2 p_1} \qquad (4)$$

where S represents the adsorption selectivity, q_1 and q_2 are the gas adsorption capacities of component 1 and 2, and p_1 and p_2 refer to the partial pressure of component 1 and 2, respectively.

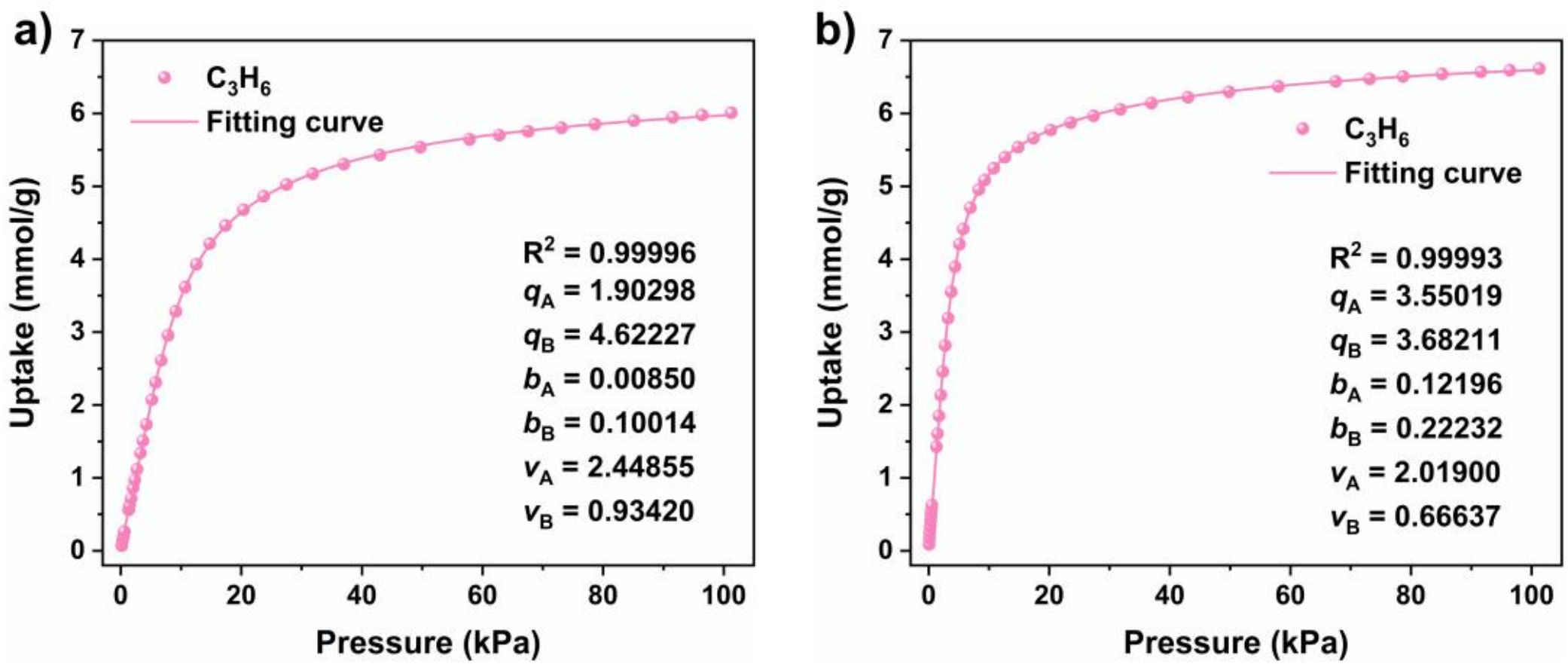

Fig. S17 The coefficients and adjusted R^2 value obtained from fitting the C_3H_6 isotherms of NKU-400 at (a) 298 K and (b) 273 K to the DSLF model.

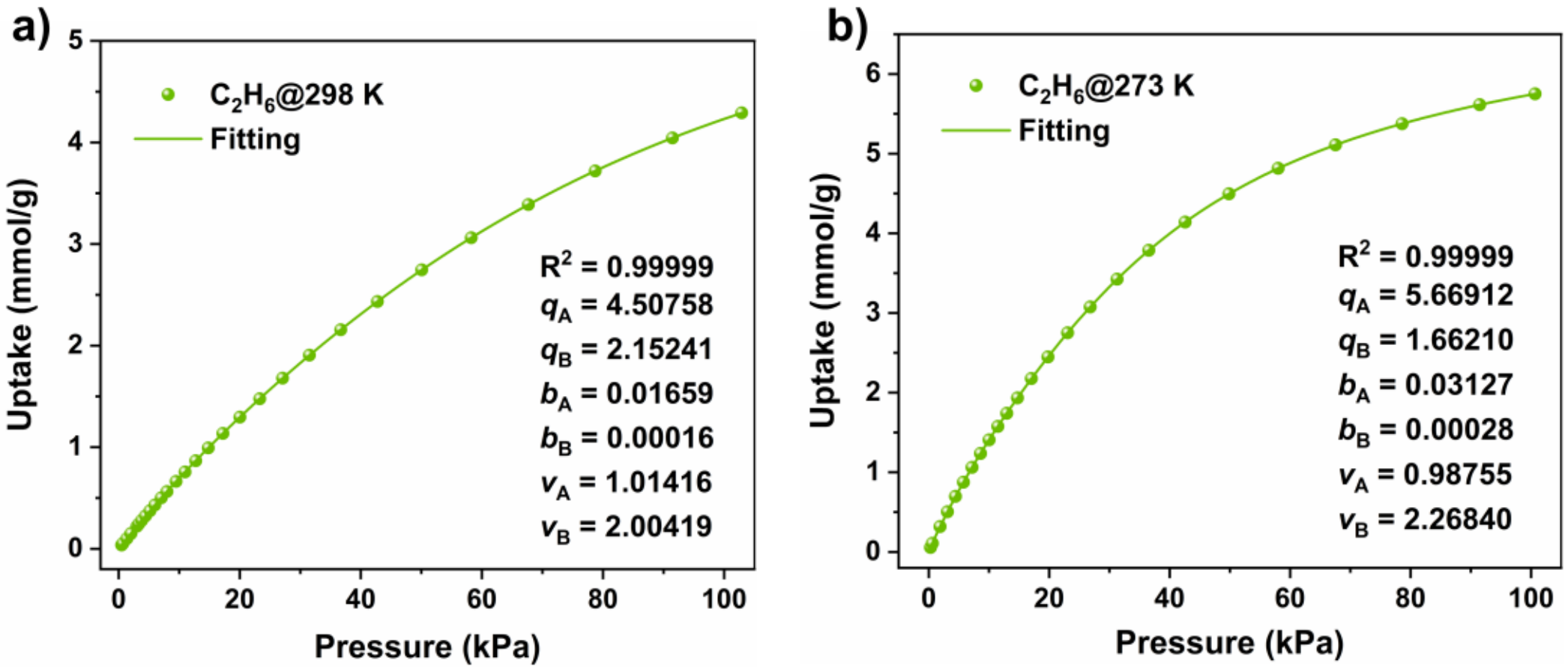

Fig. S18 The coefficients and adjusted R^2 value obtained from fitting the C_2H_6 isotherms of NKU-400 at (a) 298 K and (b) 273 K to the DSLF model.

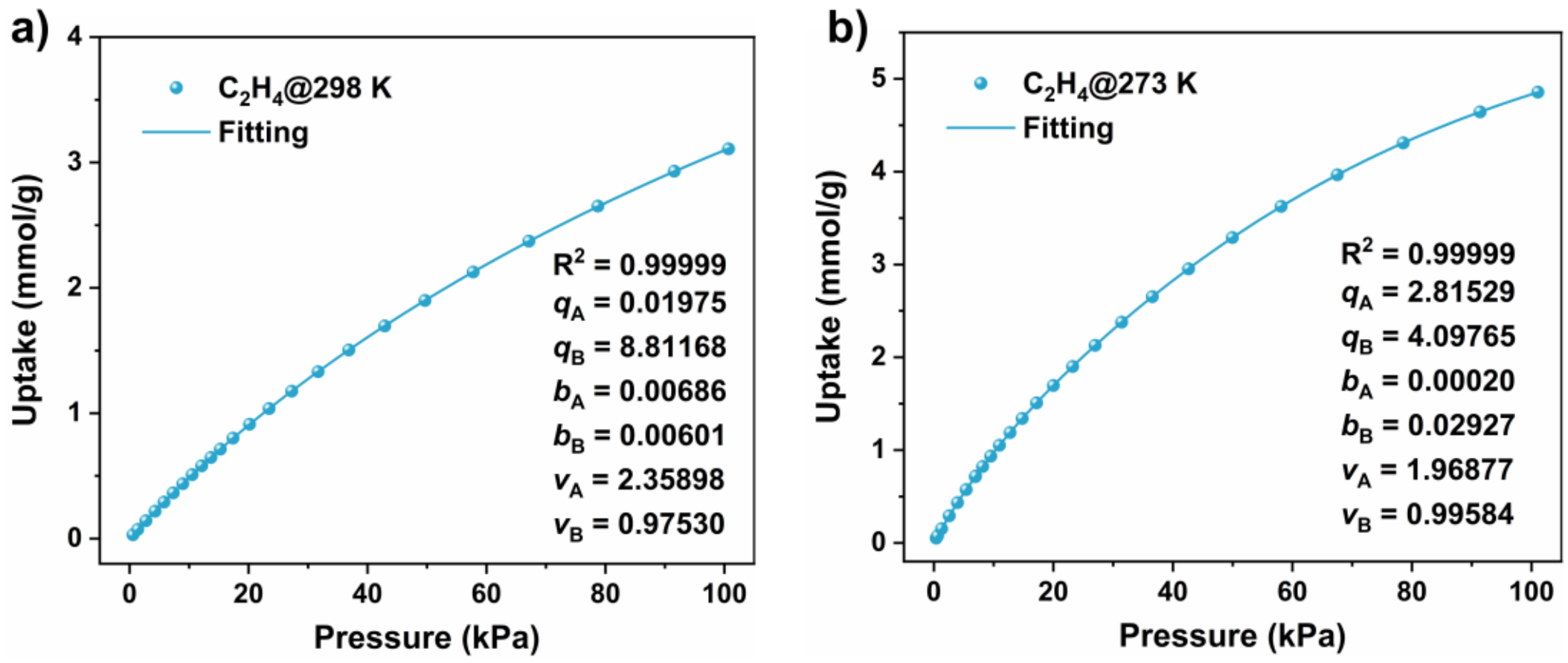

Fig. S19 The coefficients and adjusted R^2 value obtained from fitting the C_2H_4 isotherms of NKU-400 at (a) 298 K and (b) 273 K to the DSLF model.

Table S5. Dual-sites Langmuir-Freundlich model (DSLF) fitting parameters for NKU-400.

	C_3H_6		C_2H_6		C_2H_4	
	298 K	273 K	298 K	273 K	298 K	273 K
q_A	1.90298	3.55019	4.50758	5.66912	0.01975	2.81529
q_B	4.62227	3.68211	2.15241	1.66210	8.81168	4.09765
b_A	0.00850	0.12196	0.01659	0.03127	0.00686	0.00020
b_B	0.10014	0.22232	0.00016	0.00028	0.00601	0.02927
v_A	2.44855	2.01900	1.01416	0.98755	2.35898	1.96877
v_B	0.93420	0.66637	2.00419	2.26840	0.97530	0.99584
R^2	0.99996	0.99993	0.99999	0.99999	0.99999	0.99999

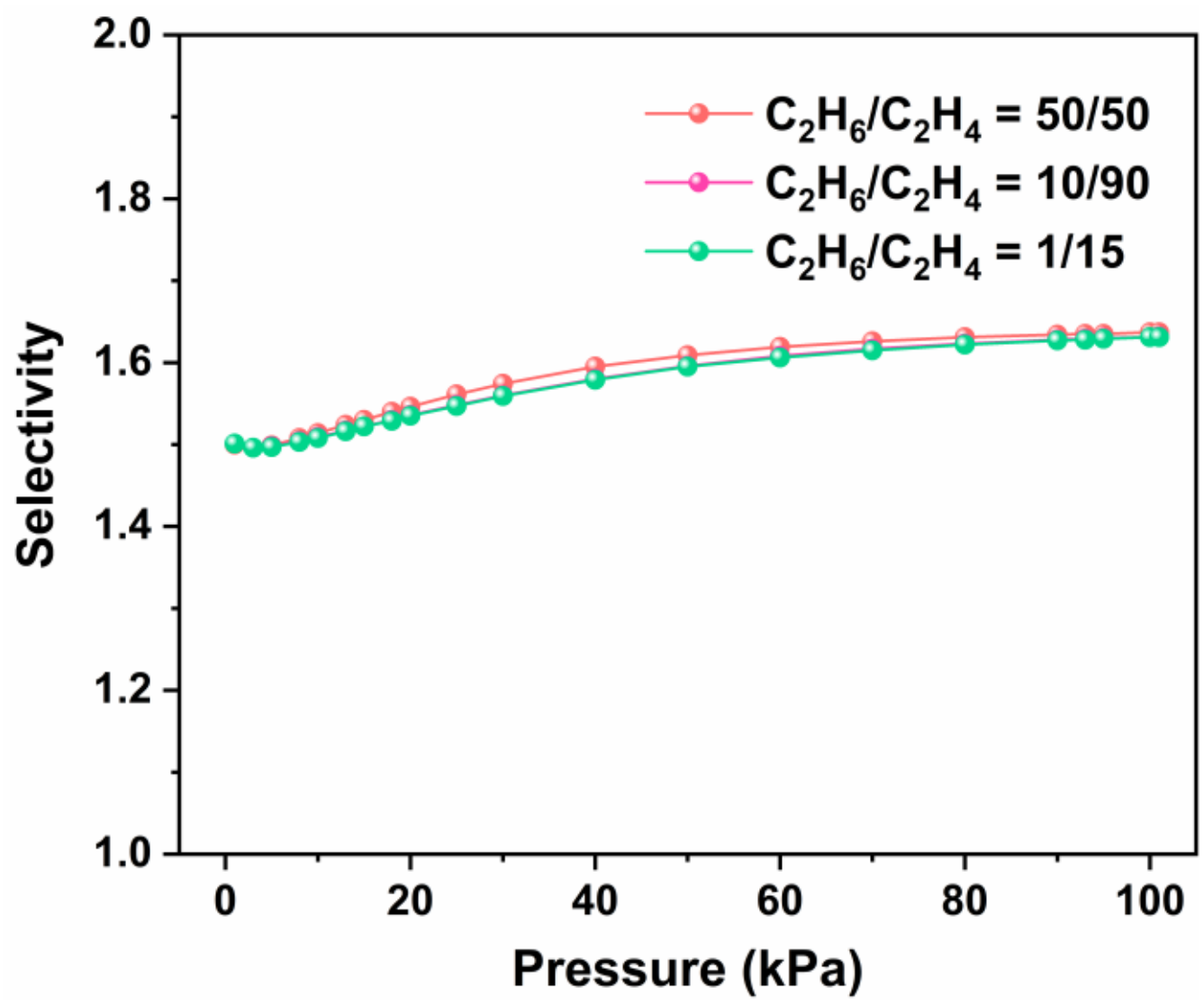

Fig. S20 IAST selectivity of C_2H_6/C_2H_4 (50/50, 10/90, 1/15; v/v) at 273 K.

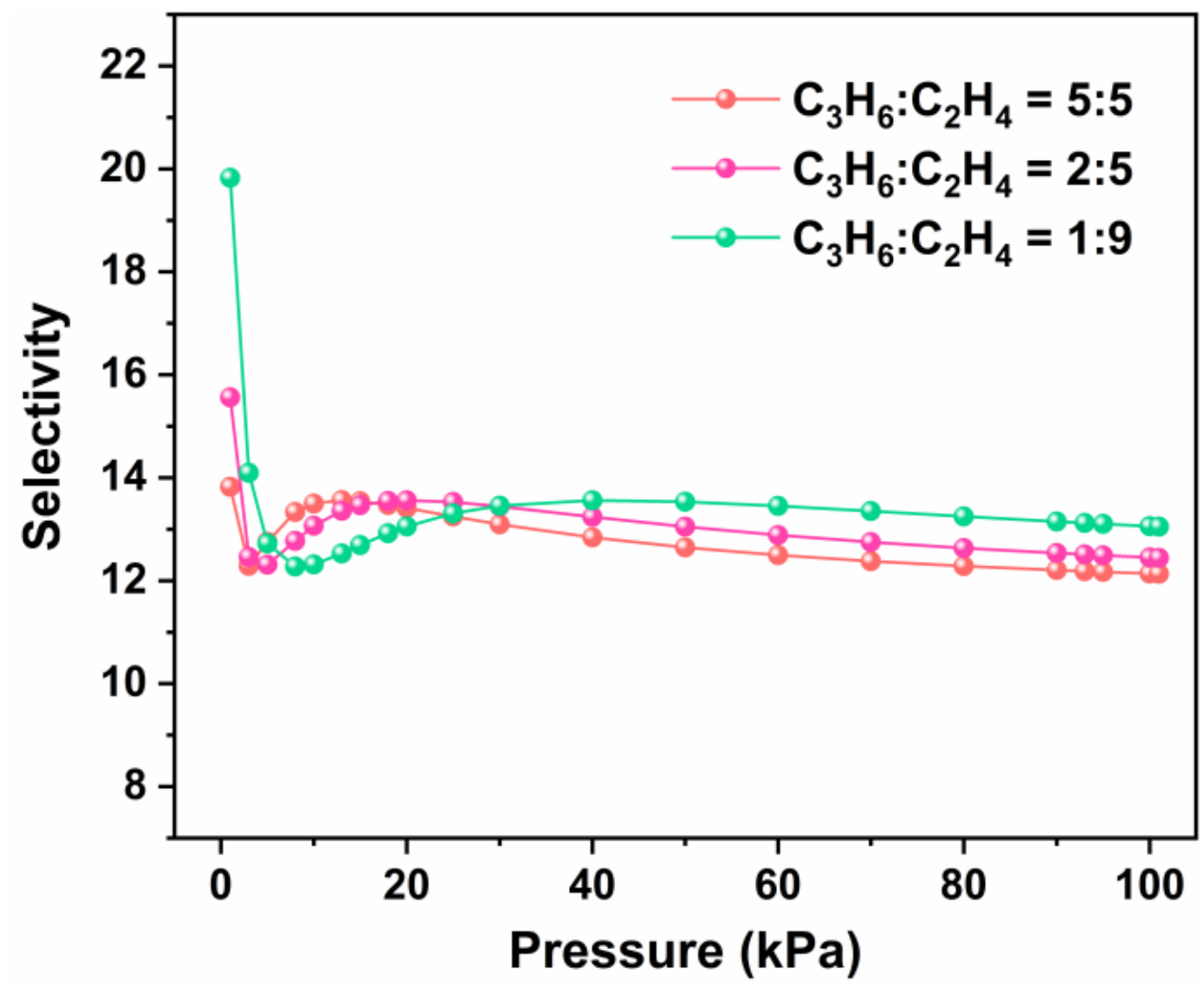

Fig. S21 IAST selectivity of C_3H_6/C_2H_4 (5/5, 2/5, 1/9; v/v) at 273 K.

Table S6. Summary of adsorption and separation metrics of some leading C_3H_6-selective MOFs at 298 K and 1 bar.

Adsorbents	C_3H_6/C_2H_4 selectivity (1/1, v/v)	C_3H_6 uptake (mmol/g)	References
Zn-BPZ-TATB	7.4	4.73	18
Quin-Zn-Ad-BTC	9.73	5.15	19
[$Zn_2(oba)_2$(dmimpym)]	17.1	3.39	20
Zn-BPZ-SA	4.8	3.05	4
spe-MOF	7.45	7.7	13
MAC-4	9.5	5.7	21
iso-MOF-4	7.74	11.36	22
$Cu_3(OH)_2(Me_2BPZ)_2$	7.4	6.16	5
MFM-202	8.4	4.53	23
CoV-bco-tpt	7.83	4.82	24
JNU-74a	13	4.22	12
FJI-H8-Me	9.9	9.42	25
Mn-dtzip	9.66	8.6	26
UPC-33	5.7	5.09	27
NEM-7-Cu	8.61	3.37	28
HOF-NBDA	11.1	5.3	29
HOF-ZJNU-3	3	5.73	30
ZJNU-401	15.45	4.53	6
ZJU-74a-Pd	23.4	4.84	31
Al-CDC	16.3	2.26	32
Al-Fum	15.7	3.66	33
SIFSIX-Cu-TPA	5.3	5.75	14
HOF-ZJNU-10	9.0	9.41	34
NKU-400	9.83	6.01	**This work**

Calculation of Separation potential (Δ*Q*)

The separation potential (Δ*Q*) is a combined metric incorporating both uptake capacity and selectivity[35, 36]. It is defined to evaluate the separation performances in fixed bed adsorbers for gas mixtures. For separation of C_3H_6/C_2H_4 mixtures, the maximum productivity of purified C_2H_4 that is theoretically achievable in a fixed bed adsorber is determined by equation 5:

$$\Delta Q = Q_1 \frac{y_2}{y_1} - Q_2 \qquad (5)$$

where Q_1 and Q_2 are C_3H_6 and C_2H_4 uptake in the mixtures, which are calculated based on IAST theory. y_1 and y_2 are the mole fractions of C_3H_6 and C_2H_4.

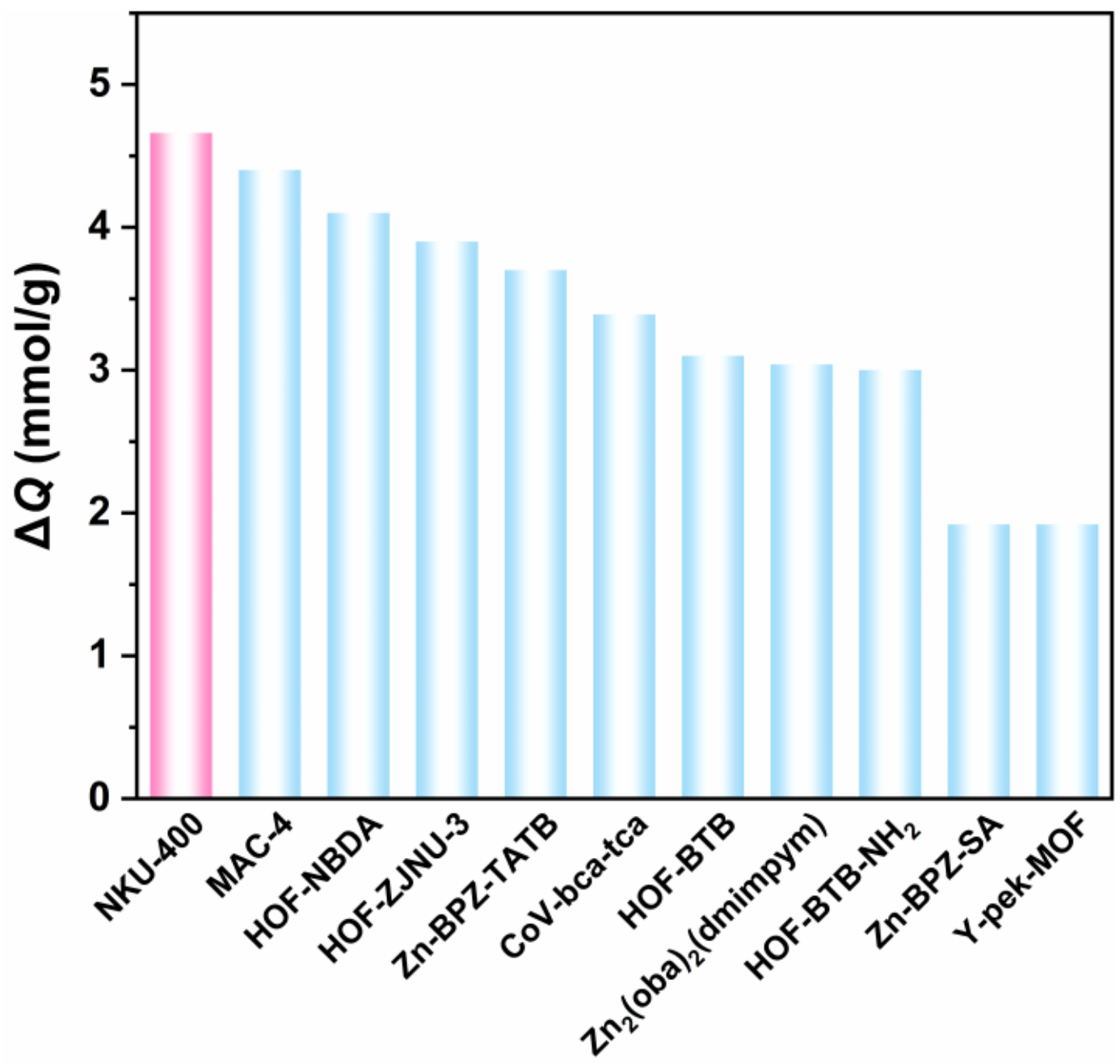

Fig. S22 Separation potential of NKU-400 and reported materials for equimolar C_3H_6/C_2H_4 mixtures at 298 K.

Section S7: Dynamic breakthrough experiments

Calculation of the breakthrough-zone width and mass-transfer-zone length

The temporal breakthrough-zone width and apparent mass-transfer-zone (MTZ) length were estimated from the corresponding single-component breakthrough curves according to a previously reported method.[37] The breakthrough time (t_b) was defined as the time at which the normalized outlet concentration (C/C_0) reached 0.01, while the equilibrium time (t_{eq}) was defined as the time at which C/C_0 reached 0.90. The values of t_b and t_{eq} were determined by linear interpolation between the adjacent experimental data points. The temporal breakthrough-zone width was calculated according to Equation:

$$\Delta t_{1-90} = t_{\mathrm{eq}} - t_b$$

The apparent MTZ length was calculated using Equation:

$$L_{MTZ} = H \frac{t_{\mathrm{eq}} - t_b}{t_{\mathrm{eq}}}$$

where H is the height of the packed adsorbent bed, which was 58.0 mm in the present experiments.

The normalized apparent MTZ length was calculated according to Equation:

$$\frac{L_{\mathrm{MTZ}}}{H} = \frac{\left(t_{\mathrm{eq}} - t_b\right)}{t_{\mathrm{eq}}}$$

For example, the apparent MTZ length of C_3H_6 was calculated as follows:

$L_{\mathrm{MTZ}} = 58.0 \times (157.21 - 110.84)/157.21 = 17.11$ mm

A smaller $\frac{L_{\mathrm{MTZ}}}{H}$ value generally corresponds to a sharper breakthrough front and more effective utilization of the packed adsorbent bed.

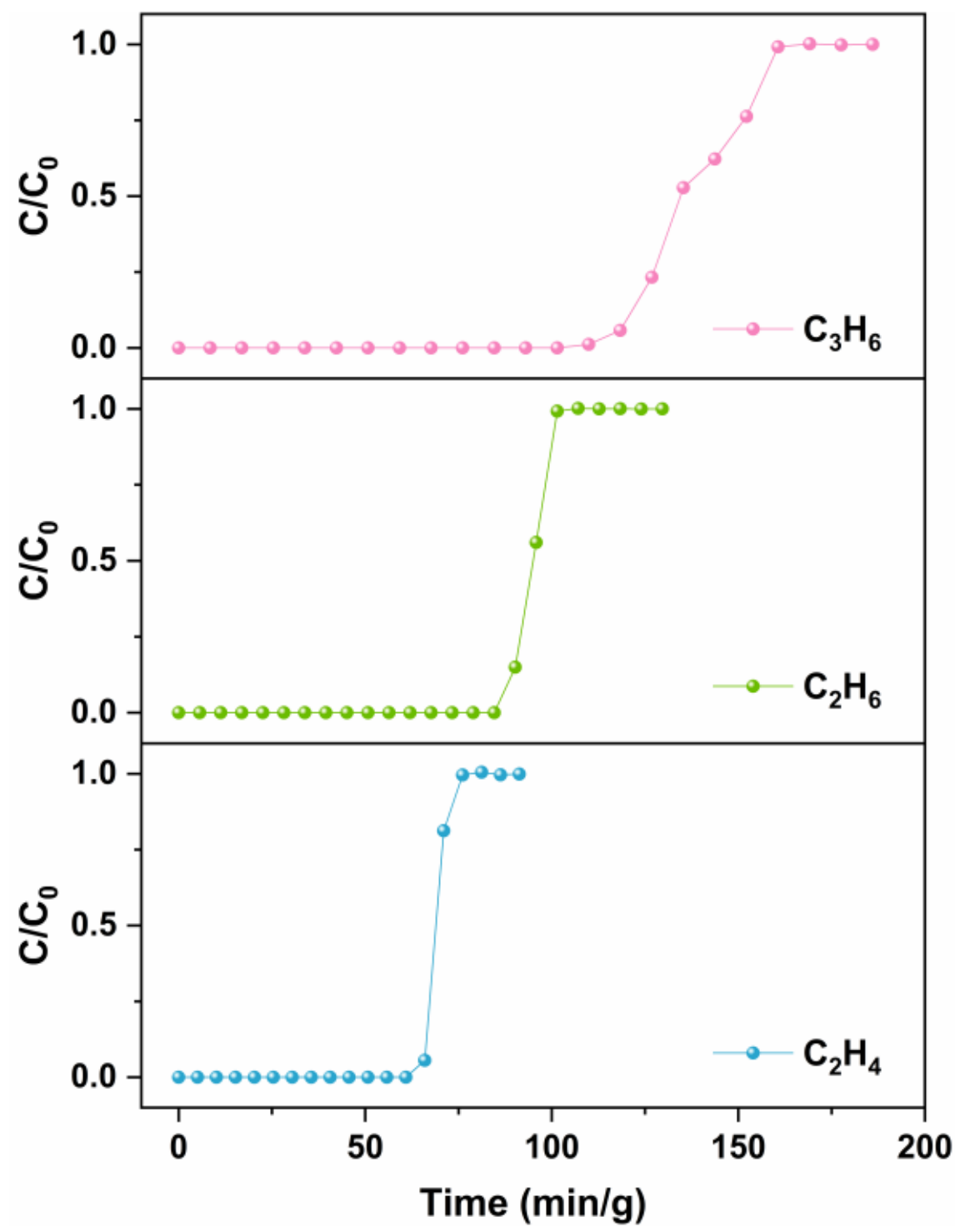

Fig. S23 Single-component breakthrough curves of C_3H_6, C_2H_6 and C_2H_4 at a flow rate of 1 mL min^{-1}.

Table S7. Temporal breakthrough-zone widths and apparent mass-transfer-zone parameters derived from the single-component breakthrough curves of C_2H_4, C_2H_6, and C_3H_6.

Gas	t_b (min g^{-1})	t_{eq} (min g^{-1})	Δt_{1-90} (min g^{-1})	L_{MTZ} (mm)	L_{MTZ}/H (%)
C_2H_4	62.10	73.46	11.36	8.97	15.4
C_2H_6	85.13	100.56	15.43	8.90	15.3
C_3H_6	110.84	157.21	46.37	17.11	29.5

The dynamic adsorption capacity (q_i) was calculated from the breakthrough curve by the equation:

$$q_i = C_i V \cdot \int_{t_0}^{t_1} (F_0(t) - F_i(t))\, dt$$

Where q_i is the equilibrium adsorption capacity of gas i (mmol g^{-1}), C_i is the feed gas concentration, V is the volumetric feed flow rate (mL min^{-1}), t is the adsorption time (min), $F_i(t)$ is the function of the breakthrough curve.

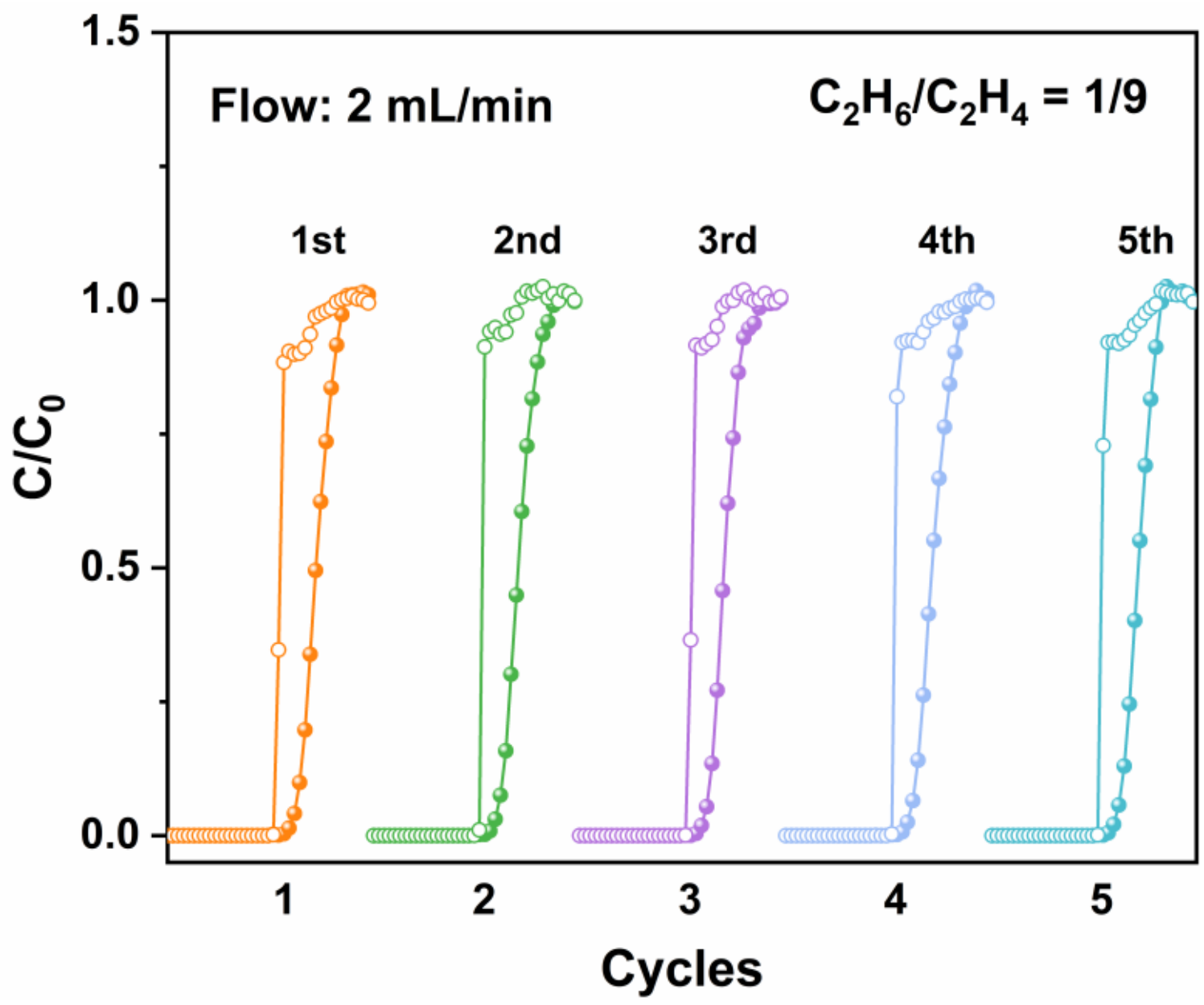

Fig. S24 Breakthrough cycles of NKU-400 for C_2H_6/C_2H_4 (1/9, v/v) mixtures at 298 K.

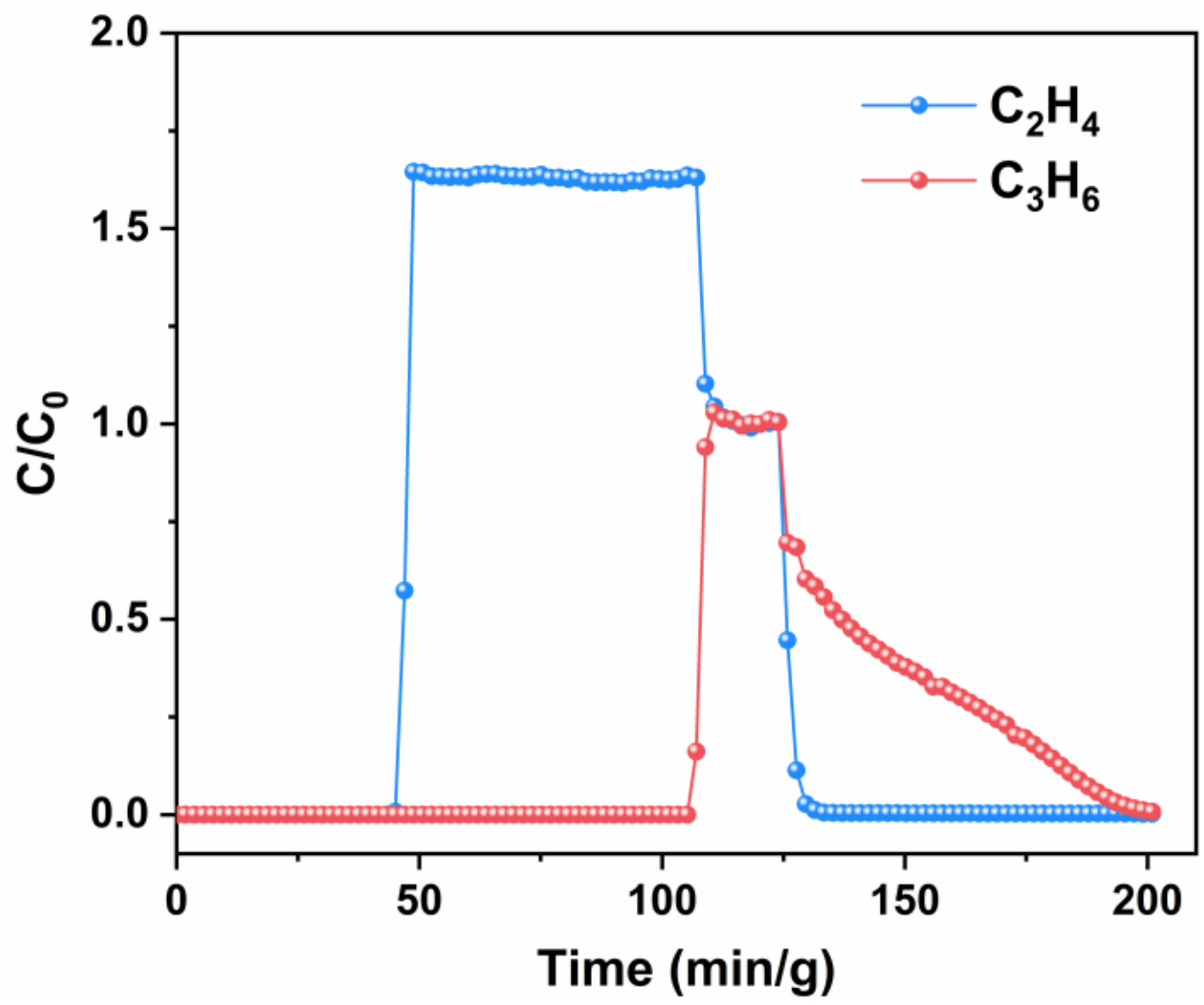

Fig. S25 Breakthrough curves for C_3H_6/C_2H_4 (1/1, v/v) at 298 K; after adsorption saturated, the desorption curves obtained under Ar (6 mL min^{-1}) sweeping at 298 K.

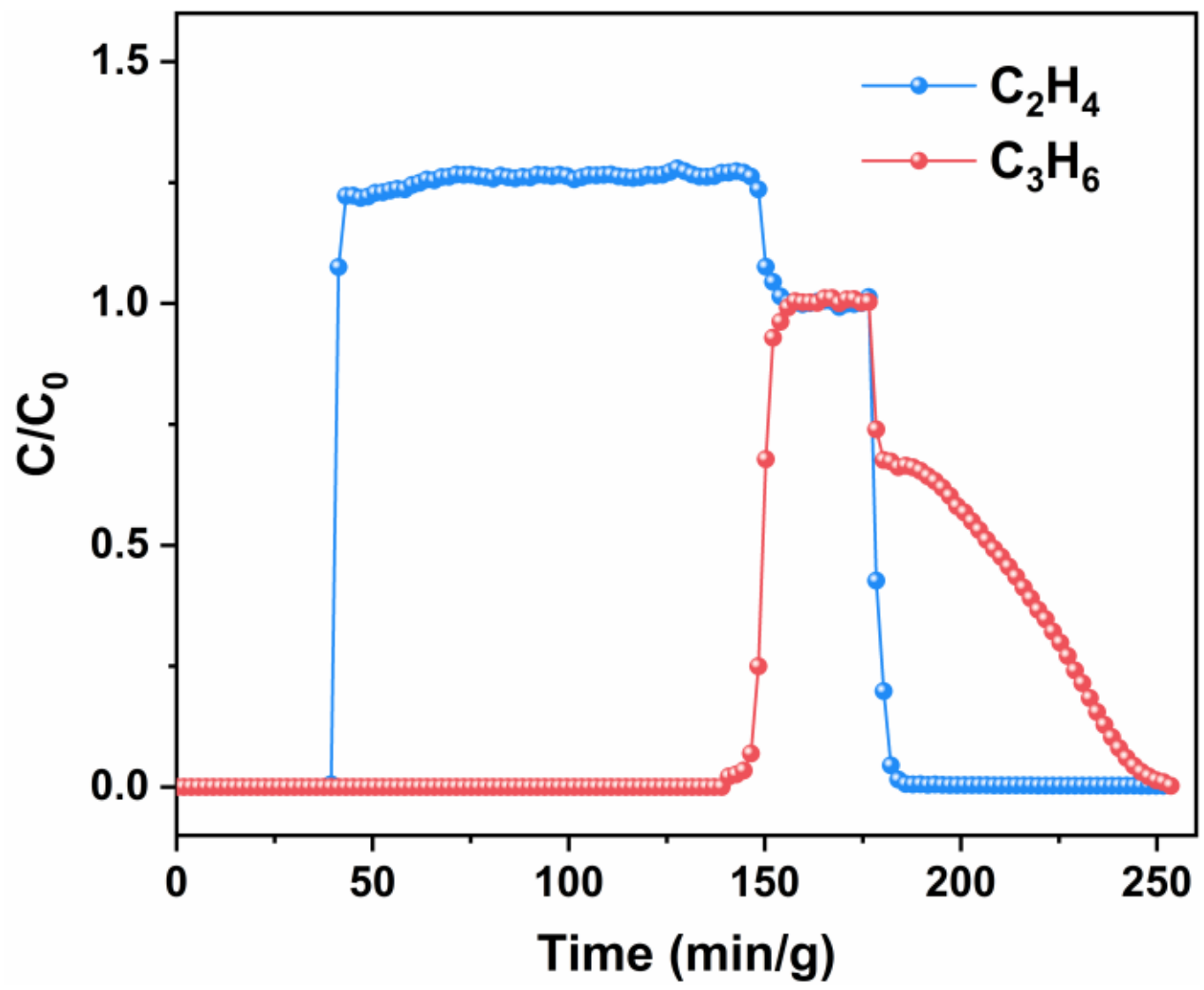

Fig. S26 Breakthrough curves for C_3H_6/C_2H_4 (2/5, v/v) at 298 K; after adsorption saturated, the desorption curves obtained under Ar (6 mL min^{-1}) sweeping at 298 K.

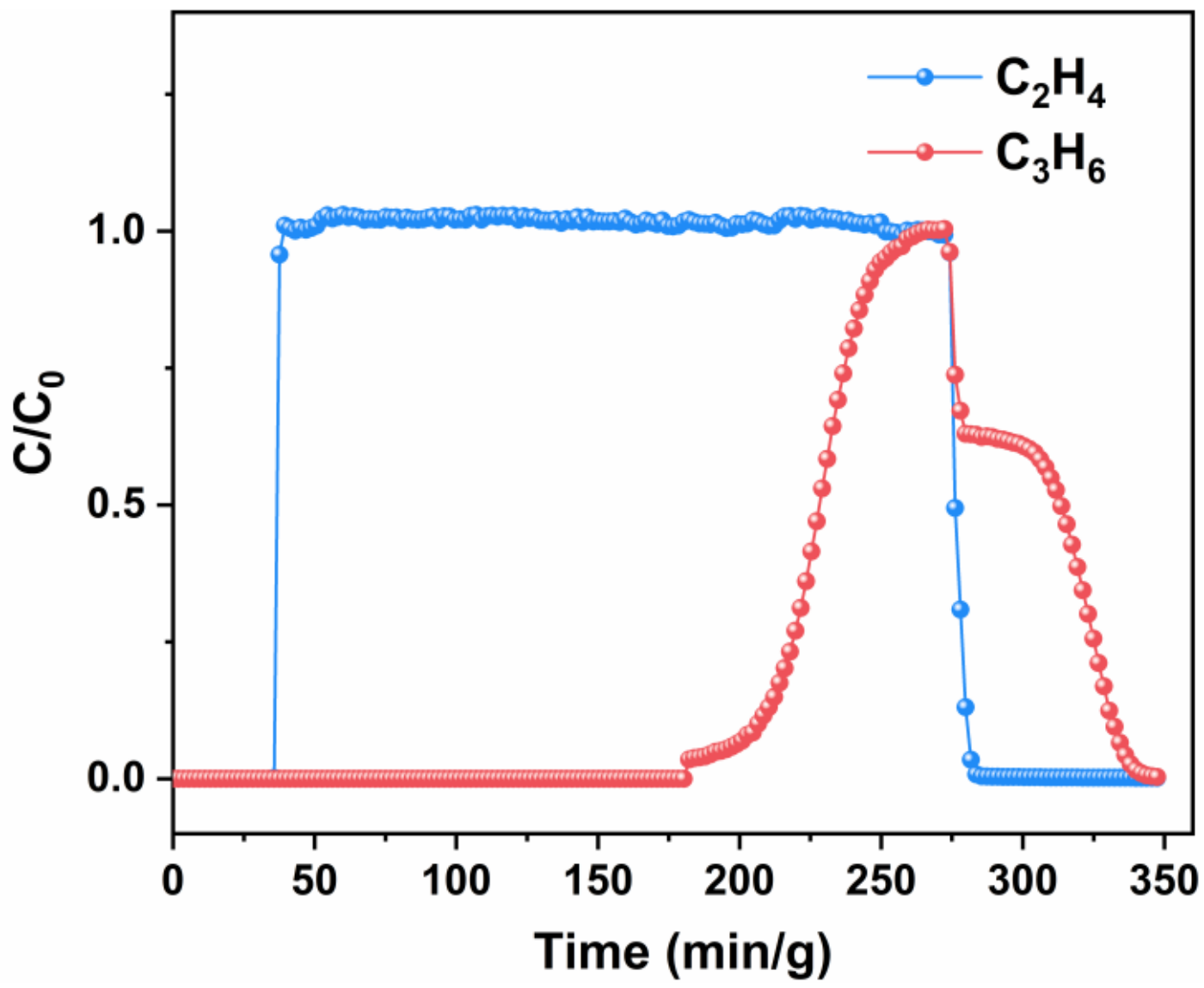

Fig. S27 Breakthrough curves for C_3H_6/C_2H_4 (1/9, v/v) at 298 K; after adsorption saturated, the desorption curves obtained under Ar (6 mL min^{-1}) sweeping at 298 K.

Table S8. Summary of high-purity C_2H_4 (≥ 99.95%) and C_3H_6 (≥ 99.5%) productivities obtained from dynamic breakthrough and subsequent desorption experiments.

Feed mixture	C_2H_4 productivity [L(STP) kg^{-1}]	C_3H_6 productivity [L(STP) kg^{-1}]
C_3H_6/C_2H_4 (1/1, v/v)	94.45	77.62
C_3H_6/C_2H_4 (2/5, v/v)	176.55	66.10
C_3H_6/C_2H_4 (1/9, v/v)	263.68	33.84
$C_3H_6/C_2H_6/C_2H_4$ (1/1/1, v/v/v)	1.83	59.72
$C_3H_6/C_2H_6/C_2H_4$ (10/2/25, v/v/v)	0.51	54.07

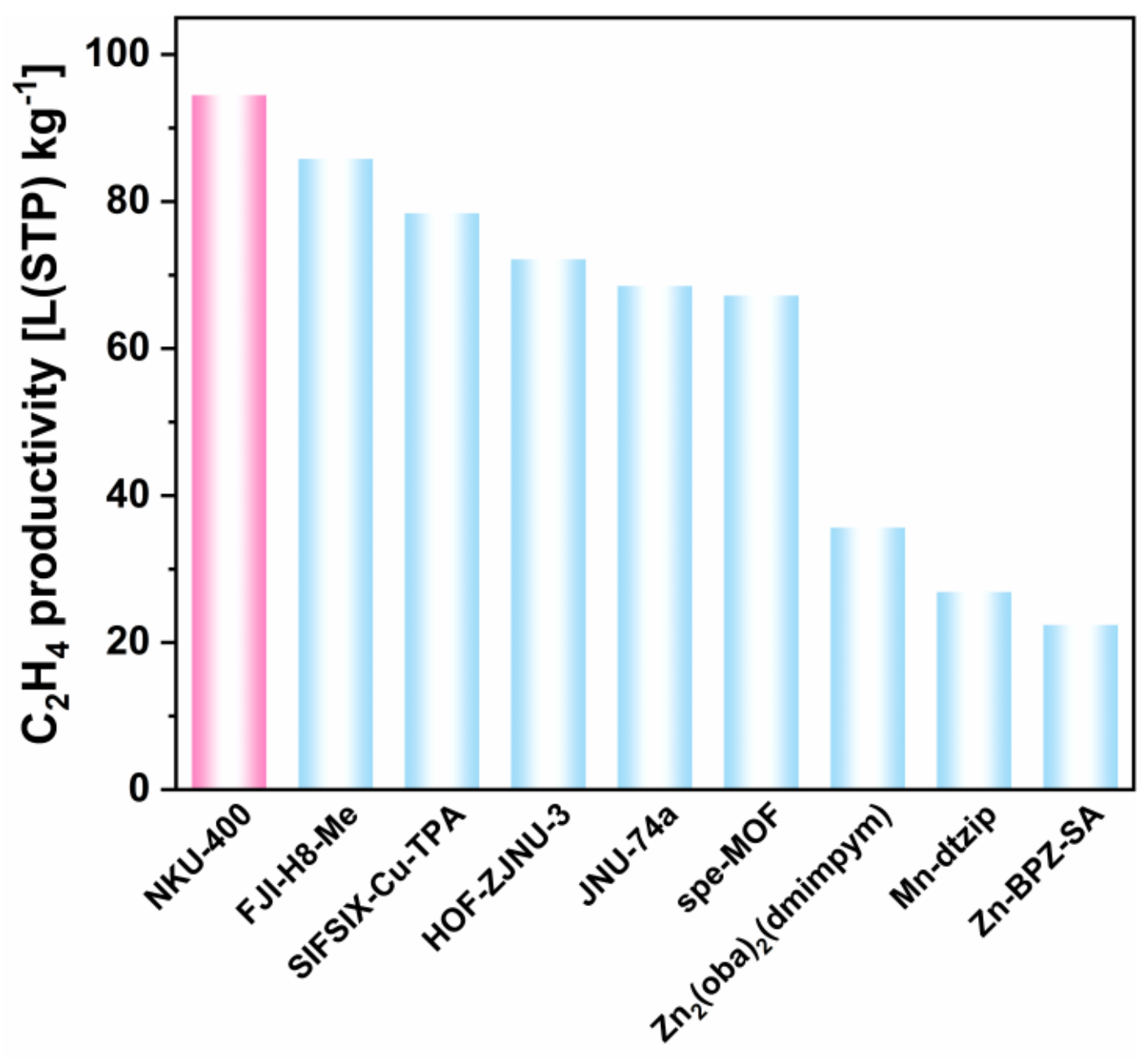

Fig. S28 Comparison of high-purity C_2H_4 (99.95%) productivities for equimolar C_3H_6/C_2H_4 (1/1, v/v) mixtures over NKU-400 and several reported adsorbents. All volumetric productivities are reported at STP (273.15 K and 1 atm).

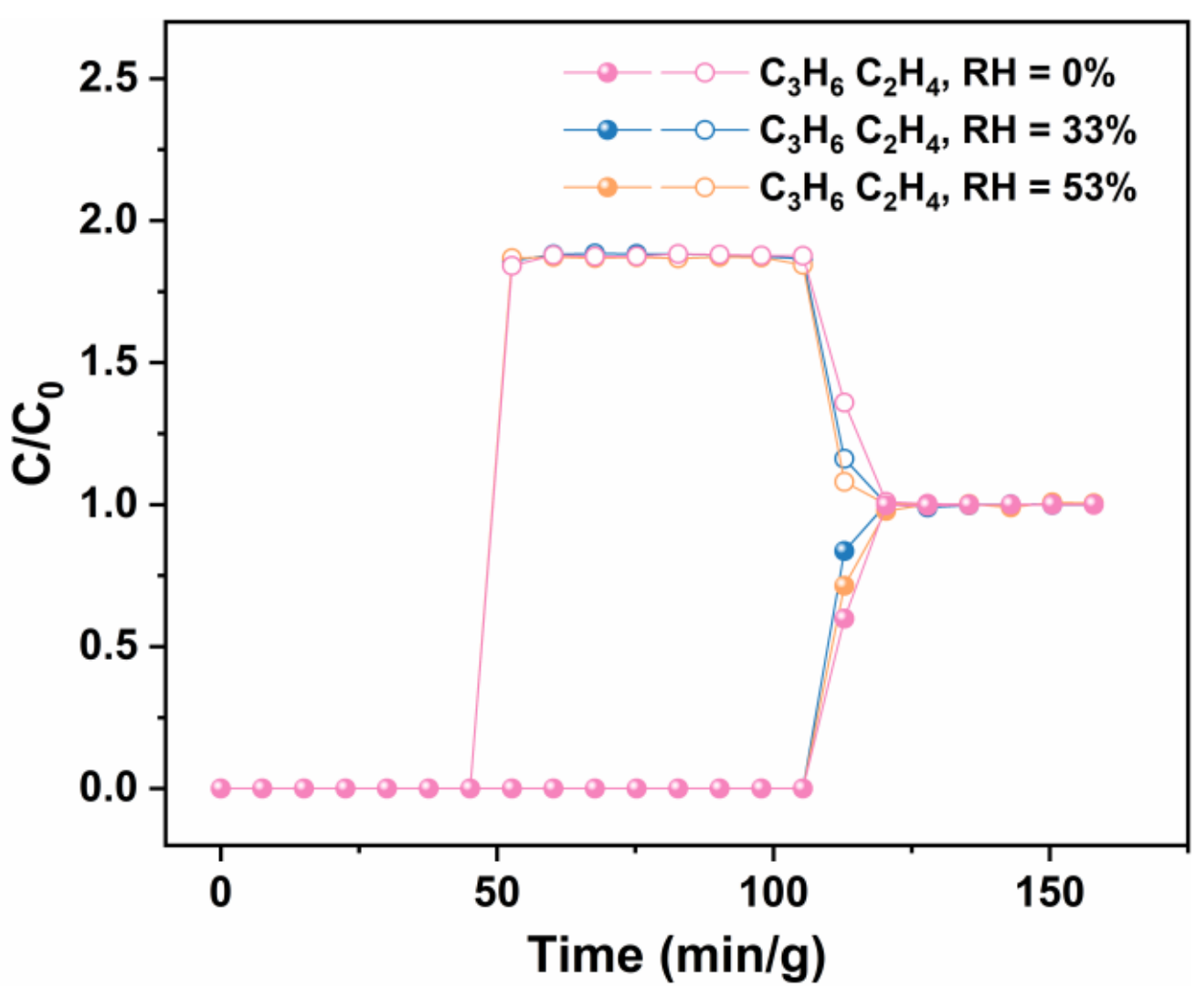

Fig. S29 Breakthrough curves for an equimolar C_3H_6/C_2H_4 (1/1, v/v) mixture on NKU-400 at 298 K under different relative humidities (RH = 0%, 33% and 53%) with a total flow rate of 2.0 mL min^{-1}.

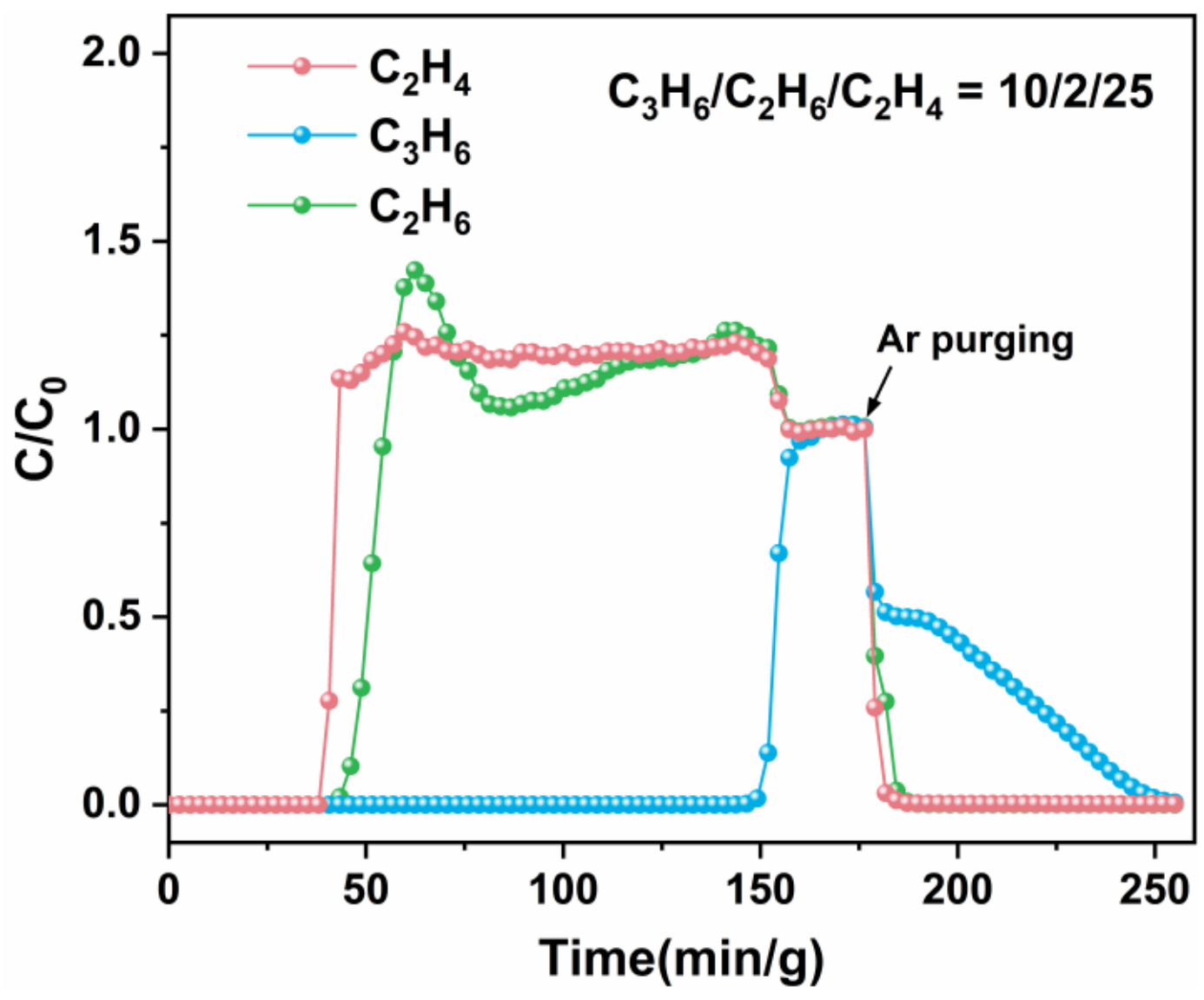

Fig. S30 Breakthrough curves for the $C_3H_6/C_2H_6/C_2H_4$ (10/2/25, v/v/v) mixtures at 298 K; after adsorption saturation, the desorption curves obtained under Ar (6 mL min^{-1}) sweeping at 298 K.

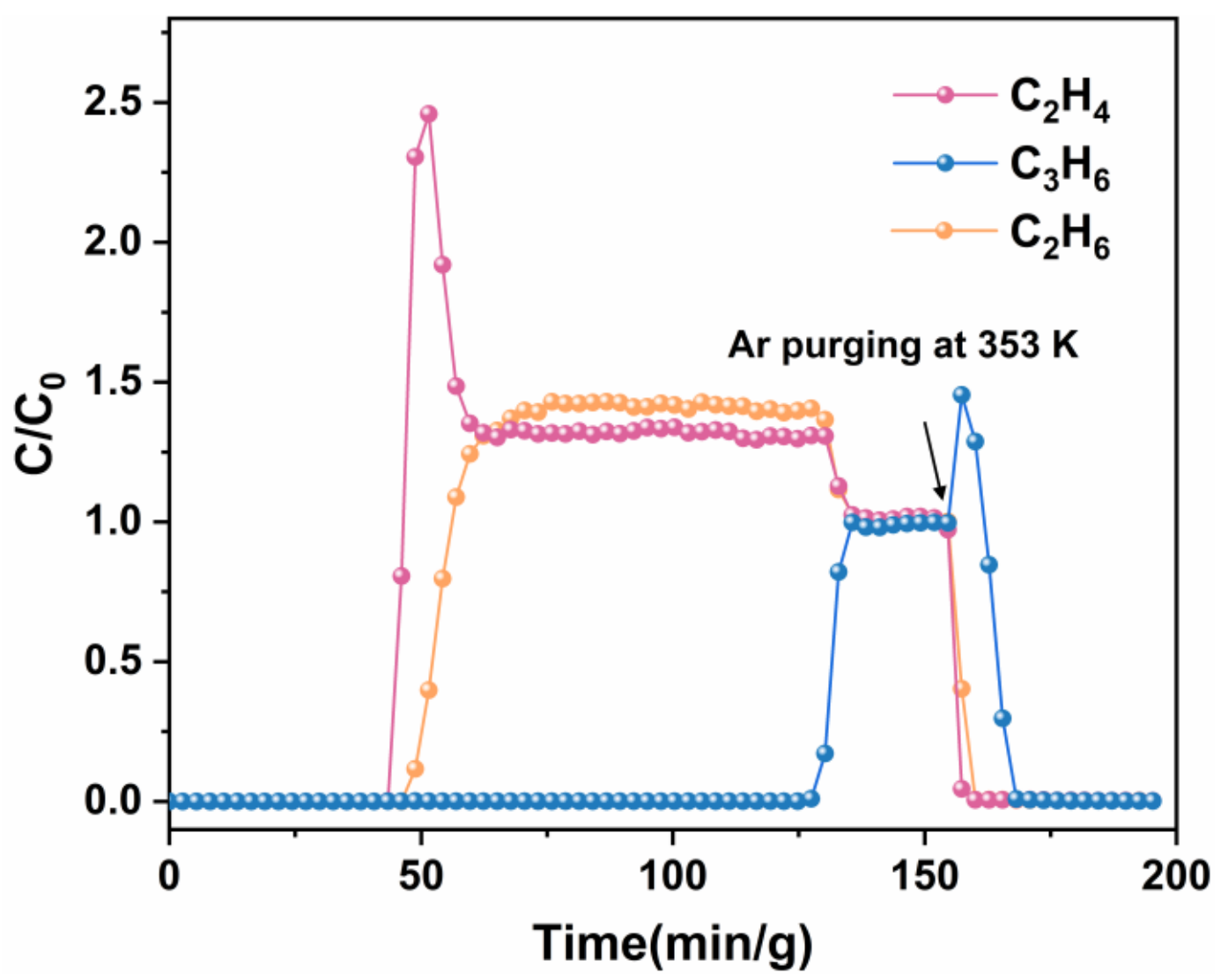

Fig. S31 Breakthrough curves for the $C_3H_6/C_2H_6/C_2H_4$ (1/1/1, v/v/v) mixtures at 298 K, followed by desorption under an Ar flow of 6 mL min^{-1} at 353 K after adsorption saturation.

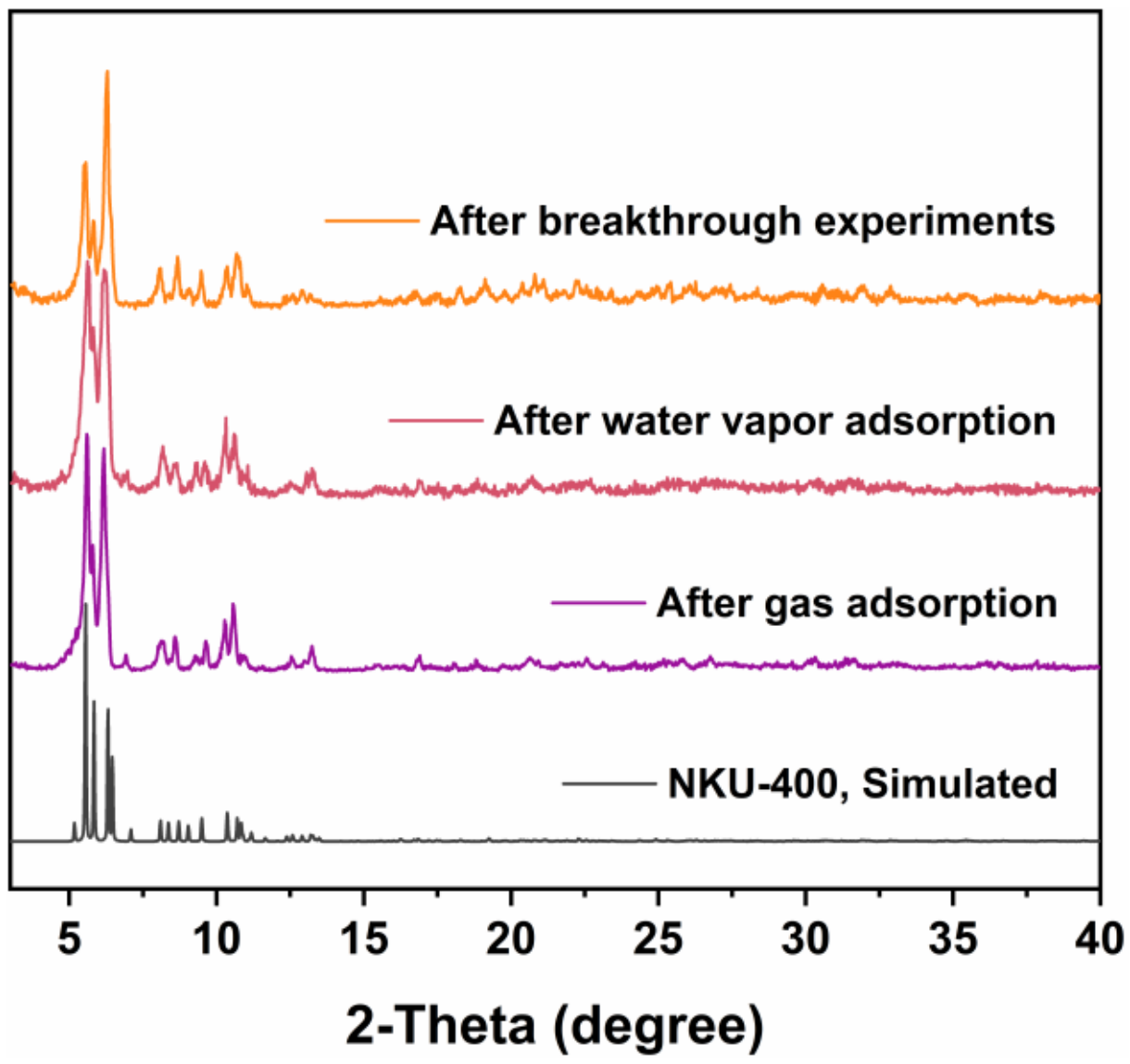

Fig. S32 Simulated PXRD pattern of NKU-400 and experimental PXRD patterns collected after gas adsorption, water-vapor adsorption, and dynamic breakthrough experiments.

Section S8: Theoretical method

Grand Canonical Monte Carlo (GCMC) simulations were performed using the Sorption module in Materials Studio to identify the preferential adsorption sites of C_3H_6, C_2H_6, and C_2H_4 in NKU-400. The Fixed Loading task was employed. The MOF framework was treated as rigid, whereas the gas molecules were geometrically optimized using the DMol^3 module[38]. Atomic partial charges were assigned by the QEq method, and intermolecular interactions were described by the Universal Force Field (UFF)[39], including Coulomb and Lennard–Jones (LJ) 12–6 potentials. Electrostatic interactions were computed using the Ewald summation method, while van der Waals interactions were calculated using an atom-based summation with a cutoff distance of 12.5 Å. Each simulation consisted of 2×10^7 steps each for loading, equilibration, production.

First-principles density functional theory (DFT) calculations with empirical dispersion corrections (DFT-D) were performed in Materials Studio to evaluate the binding energies of C_3H_6, C_2H_6, and C_2H_4 in NKU-400. The generalized gradient approximation (GGA) within the Perdew-Burke-Ernzerhof (PBE) functional was adopted, complemented by the Grimme DFT-D3(BJ) dispersion correction[40]. Electron–ion interactions were described using ultrasoft pseudopotentials. The plane-wave cutoff energy was set to 500 eV, and a Γ-centered $1 \times 1 \times 1$ k-point mesh was sampled[41]. During geometry optimization, the lattice parameters of the MOF supercell were fixed, while all atomic positions were fully relaxed until the maximum force and total energy converged to 0.05 eV Å^{-1} and 1.0×10^{-6} eV, respectively. The most probable adsorption configurations obtained from the GCMC simulations served as the initial structures for DFT optimization, followed by single-point energy calculations. The binding energy (E_b) was defined as follows:

$$E_b = E_{MOF+gas} - E_{MOF} - E_{gas}$$

where $E_{MOF+gas}$, E_{MOF}, and E_{gas} represent the total energies of the gas-adsorbed MOF system, the isolated MOF framework, and the isolated gas molecule, respectively.

Table S9. Summary of physical parameters of C_3H_6, C_2H_6 and C_2H_4.

**Gas molecules**	**C_3H_6**	**C_2H_6**	**C_2H_4**
Molecular models	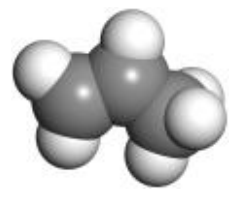	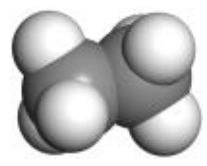	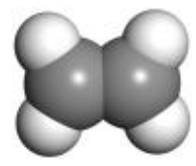
Dimensions (Å^3)	4.16 × 4.65 × 6.44	3.81 × 4.08 × 4.82	3.28 × 4.18 × 4.84
Melting point [°C]	-185	-183.3	-169.4
Boiling point [°C]	-47.7	-88.6	-103.7
Polarizability [×10^{-25} cm^3]	62.6	44.7	42.52

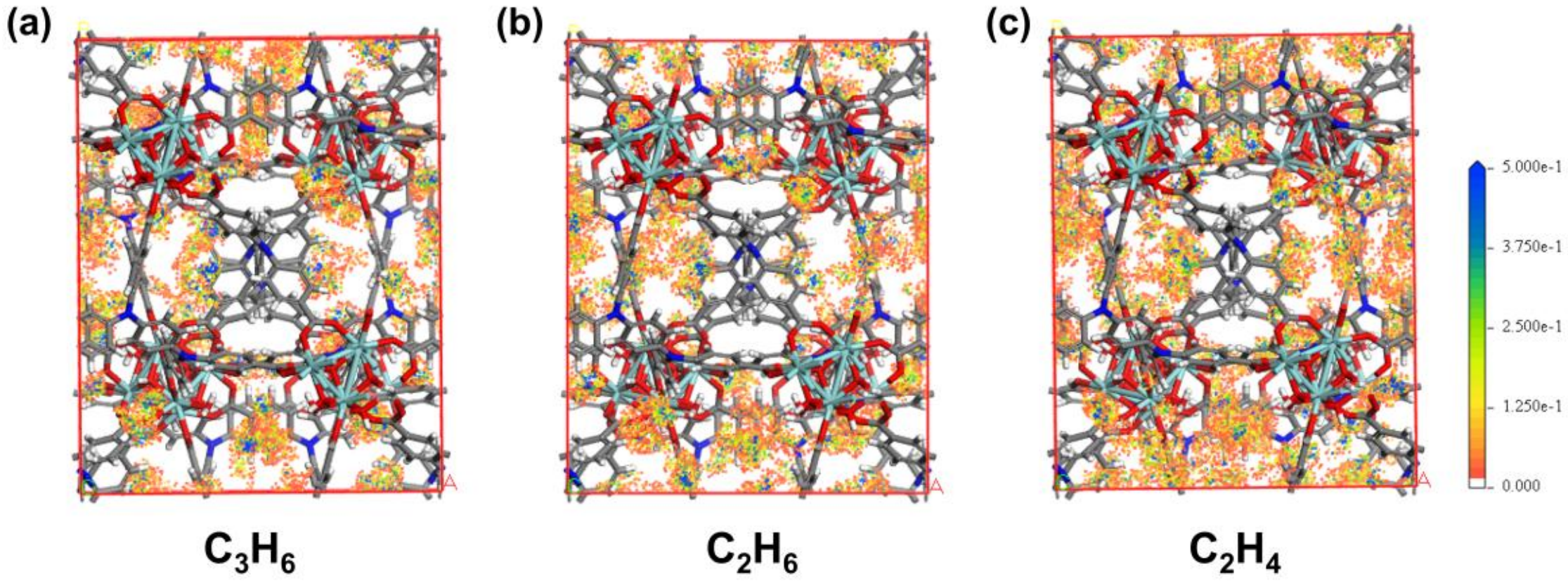

Fig. S33 The adsorption density distributions of (a) C_3H_6, (b) C_2H_6, and (c) C_2H_4 at 298 K and 100 kPa in NKU-400.

References

1 O. V. Dolomanov, L. J. Bourhis, R. J. Gildea, J. A. K. Howard and H. Puschmann, *Journal of Applied Crystallography*, 2009, **42**, 339-341.

2 G. Sheldrick, *Acta Crystallographica Section A*, 2015, **71**, 3-8.

3 G. Sheldrick, *Acta Crystallographica Section C*, 2015, **71**, 3-8.

4 G.-D. Wang, R. Krishna, Y.-Z. Li, Y.-Y. Ma, L. Hou, Y.-Y. Wang and Z. Zhu, *ACS Materials Letters*, 2023, **5**, 1091-1099.

5 G. Zhen, Y. Liu, Y. Zhou, Z. Ji, H. Li, S. Zou, W. Zhang, Y. Li, Y. Liu, C. Chen and M. Wu, *ACS Applied Materials & Interfaces*, 2023, **16**, 1179-1186.

6 J. Li, Z. Song, X. Zhou, X. Wang, M. Feng, D. Wang and B. Chen, *Chemical Science*, 2025, **16**, 7411-7417.

7 L. Lan, Q. Zhang, Y. Ren, K. Zhang, Z. Y. Qian, L. L. Wang, W. Feng, Y. L. Li, S. J. Wei, M. Feng, L. Li and T. L. Hu, *Advanced Materials*, 2026, **38**, e23278.

8 L. Yu, X. Han, H. Wang, S. Ullah, Q. Xia, W. Li, J. Li, I. da Silva, P. Manuel, S. Rudić, Y. Cheng, S. Yang, T. Thonhauser and J. Li, *Journal of the American Chemical Society*, 2021, **143**, 19300-19305.

9 Y. Huang, J. Wan, T. Pan, K. Ge, Y. Guo, J. Duan, J. Bai, W. Jin and S. Kitagawa, *Journal of the American Chemical Society*, 2023, **145**, 24425-24432.

10 Z. Deng, L. Yang, H. Xiong, J. Liu, X. Liu, Z. Zhou, S. Chen, Y. Wang, H. Wang, J. Chen, S. Deng, B. Chen and J. Wang, *Small Methods*, 2024, **9**, 2400838.

11 X. Wang, L. Bao, J.-H. Li, T. Zhao, L. Li, S. Torii, H. Chen, T. Kamiyama, P. Miao, R.-B. Lin, B. Chen and J. Gao, *Nature Communications*, 2026, **17**, 4349.

12 G. D. Wang, Y. Z. Li, H. Zeng, L. Hou, W. Lu and D. Li, *Advanced Functional Materials*, 2025, **35**, 202504916.

13 H. Fang, B. Zheng, Z. H. Zhang, H. X. Li, D. X. Xue and J. Bai, *Angewandte Chemie International Edition*, 2021, **60**, 16521-16528.

14 Z. Ji, Y. Zhou, Y. Zhu, Y. Liu, Z. Di, M. Hong and M. Wu, *ACS Materials Letters*, 2025, **7**, 837-844.

15 S.-Q. Yang, L.-L. Wang, R. Krishna, B. Xing, L. Zhou, F.-Y. Zhang, Q. Zhang, Y.-L. Li, C.-S. Bao and T.-L. Hu, *Chinese Chemical Letters*, 2025, **36**, 110556.

16 J. L. C. Rowsell and O. M. Yaghi, *Journal of the American Chemical Society*, 2006, **128**, 1304-1315.

17 A. L. Myers and J. M. Prausnitz, *AIChE Journal*, 1965, **11**, 121-127.

18 G. D. Wang, Y. Z. Li, W. J. Shi, L. Hou, Y. Y. Wang and Z. Zhu, *Angewandte Chemie International Edition*, 2023, **62**, e202311654.

19 Y. N. Ma, T. L. Liu, W. H. Zhang, C. H. He and D. X. Xue, *Angewandte Chemie International Edition*, 2025, **64**, e202513208.

20 Y.-Z. Li, G.-D. Wang, R. Krishna, Q. Yin, D. Zhao, J. Qi, Y. Sui and L. Hou, *Chemical Engineering Journal*, 2023, **466**, 143056.

21 G. D. Wang, Y. Z. Li, R. Krishna, W. Y. Zhang, L. Hou, Y. Y. Wang and Z. Zhu, *Angewandte Chemie International Edition*, 2024, **63**, e202319978.

22 W. Fan, X. Wang, X. Zhang, X. Liu, Y. Wang, Z. Kang, F. Dai, B. Xu, R. Wang and D. Sun, *ACS Central Science*, 2019, **5**, 1261-1268.

23 S. Gao, C. G. Morris, Z. Lu, Y. Yan, H. G. W. Godfrey, C. Murray, C. C. Tang, K. M. Thomas, S. Yang and M. Schröder, *Chemistry of Materials*, 2016, **28**, 2331-2340.
24 Y. Xiao, A. N. Hong, Y. Chen, H. Yang, Y. Wang, X. Bu and P. Feng, *Small*, 2022, **19**, 2205119.
25 Z. Di, Z. Ji, C. Chen, R. Krishna, D. Yuan, M. Hong and M. Wu, *Chemical Engineering Journal*, 2024, **493**, 152442.
26 L. Zhang, L.-N. Ma, G.-D. Wang, L. Hou, Z. Zhu and Y.-Y. Wang, *Journal of Materials Chemistry A*, 2023, **11**, 2343-2348.
27 W. Fan, Y. Wang, Q. Zhang, A. Kirchon, Z. Xiao, L. Zhang, F. Dai, R. Wang and D. Sun, *Chemistry – A European Journal*, 2017, **24**, 2137-2143.
28 X. Liu, C. Hao, J. Li, Y. Wang, Y. Hou, X. Li, L. Zhao, H. Zhu and W. Guo, *Inorganic Chemistry Frontiers*, 2018, **5**, 2898-2905.
29 Y. Zhou, C. Chen, Z. Ji, R. Krishna, Z. Di, D. Yuan and M. Wu, *ACS Materials Letters*, 2024, **6**, 1388-1395.
30 S. Lin, P. Ke, L. Wang, Y. Liang, J. Hu, L. Zhang, Z. Zhang, Y. He and B. Chen, *Angewandte Chemie International Edition*, 2025, **64**, e202511773.
31 J.-X. Wang, T.-F. Zhang, J. Pei, D. Liu, Y.-B. Wang, X.-W. Gu, G. Qian and B. Li, *Chem & Bio Engineering*, 2024, **1**, 952-959.
32 M. Chang, Z. Wang, R. Wang, M. Liu, Y. Wang and D. Liu, *Angewandte Chemie International Edition*, 2025, **64**, e202515496.
33 M. Chang, C. Wang, S. Song, R. Wang, J. Ma, C. Yang, Y. Wang and D. Liu, *Chemical Engineering Journal*, 2026, **530**, 173789.
34 S. Lin, Y. Qiao, Y. Du, Y. Wang, Y. Xie, Y. He, L. Li and B. Chen, *Angewandte Chemie International Edition*, 2026, **65**, e4395812.
35 R. Krishna, *RSC Advances*, 2017, **7**, 35724-35737.
36 R. Krishna, *ACS Omega*, 2020, **5**, 16987-17004.
37 E. Grigor, J. Carver, E. Bulan, S. Scott, Y. J. Chew and S. Perera, *Advanced Science*, 2024, **11**, 2406551.
38 B. Delley, The Journal of Chemical Physics, 2000, **113**, 7756-7764.
39 A. K. Rappe, C. J. Casewit, K. S. Colwell, W. A. Goddard, III and W. M. Skiff, Journal of the American Chemical Society, 1992, **114**, 10024-10035.
40 J. P. Perdew, K. Burke and M. Ernzerhof, Physical Review Letters, 1996, **77**, 3865-3868.
41 H. J. Monkhorst and J. D. Pack, Physical Review B, 1976, **13**, 5188-5192.
